# Molecular Mechanisms and Therapeutic Potential of DJ‐1 in Skeletal Muscle Homeostasis and Disease

**DOI:** 10.1002/cph4.70091

**Published:** 2026-02-01

**Authors:** Yue Zhang, Menghuan Li, Xiaojing Xie, Baokai Tian, Zhenwei Bao, Xuejie Yi

**Affiliations:** ^1^ School of Sports and Health Shenyang Sport University Shenyang China; ^2^ School of Physical Education Liaoning Normal University Dalian China; ^3^ Sports and Health Research Center Shenyang Sport University Shenyang China

**Keywords:** DJ‐1, mitochondria, muscle diseases, oxidative stress, skeletal muscle

## Abstract

The DJ‐1 protein was initially identified as an oncogene, but subsequent studies revealed its crucial protective role in neurodegenerative diseases. Increasing evidence indicates that DJ‐1 possesses critical physiological functions in skeletal muscle, but the underlying mechanisms remain to be systematically elucidated. Existing research has conclusively demonstrated that DJ‐1 is widely expressed in skeletal muscle and functions as a central hub integrating multiple pathways to establish a multi‐level cellular protection network: it safeguards energy metabolism by maintaining mitochondrial structure and function; enhances antioxidant capacity by directly scavenging ROS and regulating Nrf2/ARE signaling; and delays muscle aging by inhibiting protein aggregation and preserving protein homeostasis. Under pathological conditions, DJ‐1 dysfunction is closely associated with muscular dystrophy, inflammatory myopathy, metabolic myopathy, and muscle atrophy. Its abnormalities lead to mitochondrial damage, exacerbated oxidative stress, and disrupted protein homeostasis, ultimately triggering muscle structural deterioration. Based on these insights, researchers have developed various DJ‐1 regulatory strategies, including small‐molecule activators, transcriptional modulators, and functional peptide compounds, which show promising therapeutic potential. This review represents the first systematic, cross‐disease integration of DJ‐1's role in aging‐related sarcopenia, diabetic myopathy, inflammatory myopathies, and neuromuscular degenerative diseases. It elucidates DJ‐1's core function as a central integrator coordinating antioxidant defense, mitochondrial homeostasis, metabolic regulation, and protein homeostasis. A deeper understanding of DJ‐1's mechanisms will provide critical theoretical foundations for elucidating the common pathological basis of skeletal muscle diseases and developing novel therapeutic strategies.

## Graphical abstract

DJ‐1 protein functions as a redox‐sensing guardian in skeletal muscle by coordinating antioxidant defense, mitochondrial homeostasis, metabolic adaptation, and anti‐atrophy signaling. Enhancing its function holds promise as a potential therapeutic strategy for muscle atrophy, ALS, and metabolic myopathies.

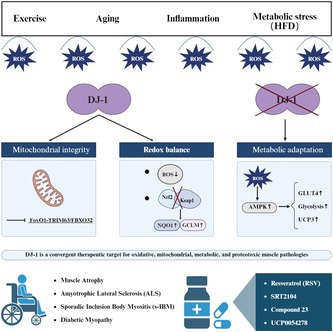

## 1 Introduction

Skeletal muscle, comprising approximately 40% of body weight, represents humans' most significant metabolic organ and plays a central role in locomotion, glucose homeostasis, and whole‐body energy balance (Groeneveld 2024). With global population aging, sarcopenia affects over 50 million people worldwide, with healthcare costs reaching $18.5 billion in the United States alone in 2000 (Chen et al. 2020; Janssen et al. 2004). Sarcopenia is classified into primary (primarily age‐related) and secondary (caused by chronic diseases, malnutrition, or inactivity) forms (Bauer et al. 2019), yet effective molecular‐targeted therapies remain limited.

The maintenance of skeletal muscle homeostasis relies on the coordinated regulation of multiple molecular networks, among which peroxisome proliferator‐activated receptor gamma coactivator 1‐alpha (PGC‐1α) and the Forkhead box O (FoxO) transcription factor family (FoxOs) are the most extensively studied core regulators. PGC‐1α serves as a primary regulator of mitochondrial biogenesis, oxidative metabolism, and anti‐inflammatory responses, yet its activity is predominantly driven by upstream metabolic signals and lacks direct sensing of oxidative stress (Abu Shelbayeh et al. 2023). FoxOs promote atrophic phenotypes by regulating the ubiquitin‐proteasome system (UPS) and autophagy pathways, but their oxidative stress response relies on multi‐step signaling, exhibiting indirectness and delay (Chen et al. 2022). In contrast, the precise maintenance of skeletal muscle homeostasis requires factors capable of simultaneously performing dual functions of “oxidative sensing and response execution” to counteract the rapid elevation of ROS during exercise and pathological states.

Against this backdrop, DJ‐1 has garnered increasing attention as a unique redox sensor and effector protein (Kahle et al. 2009). Physiological levels of reactive oxygen species (ROS) are essential for muscle adaptation, but excessive accumulation leads to myofiber damage, atrophy, and fatigue (Powers and Jackson 2008). While traditional antioxidant systems are crucial, they lack sophisticated sensing and rapid response capabilities (Forman and Zhang 2021). DJ‐1, however, senses oxidative status in real time through its key cysteine residue (Cys‐106) and activates protective pathways (Kinumi et al. 2004; Raninga et al. 2017). These include direct ROS scavenging (Taira et al. 2004), Nuclear factor erythroid 2‐related factor 2 (Nrf2) stabilization (Clements et al. 2006), maintenance of mitochondrial complex I activity (Hayashi et al. 2009), and autophagy flux regulation (McCoy and Cookson 2011). This near “frontline sensing‐immediate execution” characteristic confers DJ‐1 an irreplaceable functional role in skeletal muscle oxidative stress responses, offering a novel molecular target for muscle disease therapy.

DJ‐1 was initially discovered by Nagakubo et al. (1997) and identified as the Parkinson's disease‐causative gene *PARK7* in 2003 (Bonifati et al. 2003). As an evolutionarily highly conserved protein (Bandyopadhyay and Cookson 2004), DJ‐1 small‐molecule activators have demonstrated significant efficacy in neuroprotection studies (Kitamura et al. 2011), providing a translational foundation for therapeutic applications in muscle diseases. Recent studies have revealed DJ‐1's important role in skeletal muscle: differential expression across muscle fiber types (Roca‐Rivada et al. 2012); progressive muscle strength decline, reduced exercise endurance, and mitochondrial dysfunction in muscle‐specific DJ‐1 knockout mice (Zhang et al. 2023). In human studies, DJ‐1 expression levels positively correlate with muscle mass and function in the elderly (Zhang et al. 2023). These findings suggest that DJ‐1 may serve as a biomarker and therapeutic target for sarcopenia. Its functional role differs from both PGC‐1α, which is biased toward metabolic regulation, and FoxOs, which dominate the atrophy response. Instead, DJ‐1 focuses on the precise sensing of oxidative stress and the rapid initiation of protective responses.

However, critical knowledge gaps regarding DJ‐1's regulation of skeletal muscle function: fiber type‐specific actions, interaction networks with key regulators such as PGC‐1α/FoxOs, dynamic changes under muscle pathological states, and therapeutic target potential all require clarification. This review systematically analyzes DJ‐1's expression patterns, homeostatic regulatory mechanisms, and associations with sarcopenia, ALS, inclusion body myositis, and other diseases in skeletal muscle, aiming to provide a reference for basic research and clinical translation in this field.

## 2 Overview of DJ‐1

### 2.1 Structural and Functional Characteristics

DJ‐1 is a highly conserved multifunctional protein composed of 189 amino acids with a molecular weight of approximately 20 kDa (Hod et al. 1999). It belongs to the DJ‐1/ThiJ/PfpI superfamily (Wilson et al. 2004). Its gene is located at chromosome 1p36.2‐p36.3 (Taira et al. 2001) and is abundantly expressed in metabolically active tissues such as the kidney, heart, and skeletal muscle (Nagakubo et al. 1997).

DJ‐1 adopts a typical α/β fold conformation, forming stable homodimeric structures (Wilson et al. 2003) that are essential for its function (Tao and Tong 2003). As both an oxidative‐reductive sensor and effector protein, DJ‐1's functional core lies in the dynamic oxidative modification mechanism of the critical cysteine residue Cys‐106 (Blackinton et al. 2009). Cys‐106 undergoes progressive oxidation from thiol (‐SH) to sulfinic acid (‐SO_2_H), with moderate oxidation‐formed sulfinic acid serving as a molecular switch to activate DJ‐1's protective functions (Kinumi et al. 2004; Blackinton et al. 2009; Wilson 2011). Other cysteine residues (Cys‐46/Cys‐53) protect Cys‐106 from excessive oxidation through intramolecular disulfide bond formation (Song et al. 2021), making DJ‐1 a highly sensitive “molecular sensor” for cellular oxidative stress.

### 2.2 Comparison of DJ‐1 With Other Oxidative‐Reductive Proteins

Unlike classical antioxidant enzymes (SOD, Catalase, GPx), DJ‐1 possesses both direct ROS scavenging ability and transcriptional regulatory and signal transduction functions (Neves et al. 2022; Dolgacheva et al. 2019). Traditional antioxidant enzymes primarily target single ROS species without participating in other protective pathway regulation. At the same time, DJ‐1 can scavenge multiple ROS types, stabilize Nrf2 transcription factor to upregulate antioxidant enzyme expression (Clements et al. 2006; Yang et al. 2017; Moscovitz et al. 2015), and regulate PI3K/Akt signaling pathways (Tanti and Goswami 2014; Zhang et al. 2016), achieving comprehensive protection from defense to repair.

The multifunctional role of DJ‐1 in skeletal muscle is particularly crucial. Unlike other antioxidant proteins that decline steadily with age and pathological conditions, DJ‐1 can be rapidly induced under acute stress (Taira et al. 2004), forming a “first‐response” defense line. Conversely, in chronic pathological states, its function becomes impaired due to excessive oxidation or misfolding (Ito et al. 2006). This dynamic shift suggests a potential therapeutic window for targeting DJ‐1.

## 3 DJ‐1 Function and Regulatory Mechanisms in Skeletal Muscle

**FIGURE 1:**
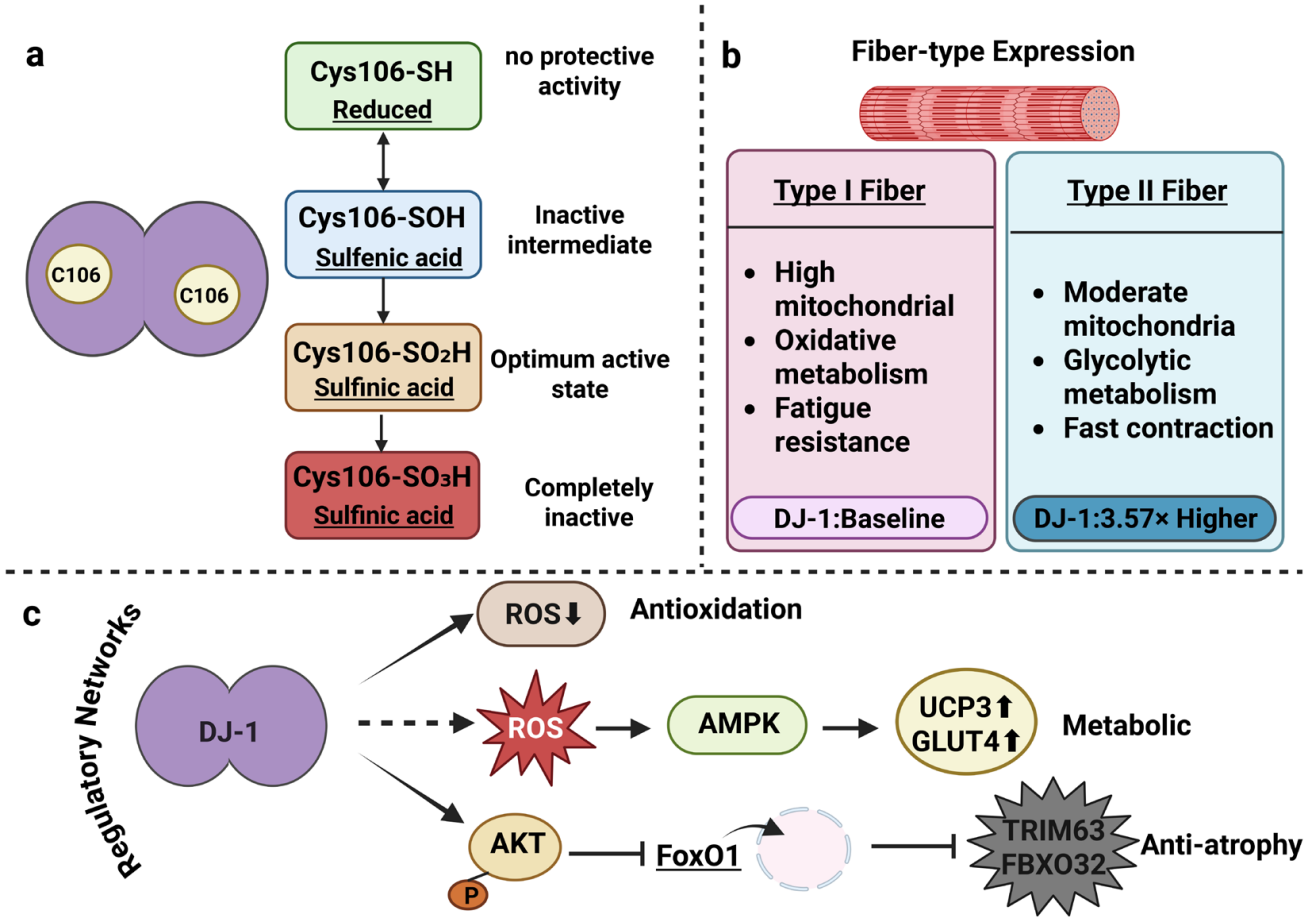
DJ‐1 structure–function relationship and regulatory networks in skeletal muscle. (a) DJ‐1 exists as a homodimer, with Cys106 serving as a critical redox‐sensitive residue. The functional activity of DJ‐1 is determined by the oxidation state of Cys106: the reduced form (Cys106‐SH) lacks cytoprotective activity; the sulfenic acid form (Cys106‐SOH) represents a transient intermediate with limited activity; oxidation to the sulfinic acid state (Cys106‐SO₂H) confers optimal functional activity; whereas further oxidation to the sulfonic acid form (Cys106‐SO₃H) results in irreversible inactivation. (b) DJ‐1 expression displays fiber‐type specificity in skeletal muscle. Type II fibers exhibit approximately 3.57‐fold higher DJ‐1 expression compared with Type I fibers; however, Type I fibers demonstrate greater sensitivity to DJ‐1 deficiency, indicating a stronger functional dependence on DJ‐1‐mediated redox regulation. (c) DJ‐1 maintains skeletal muscle homeostasis through three interconnected regulatory pathways: (1) antioxidant defense, via direct scavenging of reactive oxygen species (ROS); (2) metabolic adaptation, whereby DJ‐1 deficiency leads to ROS accumulation, activation of AMPK signaling, and subsequent upregulation of UCP3 and GLUT4 to promote glycolytic metabolism; and (3) anti‐atrophy signaling, in which DJ‐1 activates the AKT–FoxO1 axis to suppress the expression of muscle atrophy‐related genes, including TRIM63 and FBXO32. Created in https://BioRender.com.

Although significant progress has been made in DJ‐1 skeletal muscle function research, controversies and paradoxes remain regarding its precise mechanisms. These include DJ‐1's high expression in fast‐twitch fibers yet seemingly stronger protection for slow‐twitch fibers, marked differences in antioxidant effects across cell types, and complex functional redundancy and compensatory mechanisms. Nevertheless, extensive research has revealed DJ‐1's core role in skeletal muscle homeostasis maintenance. This chapter systematically describes its expression distribution and functional mechanisms, concluding with an objective discussion of current controversies to provide balanced perspectives for a comprehensive understanding of DJ‐1's skeletal muscle actions. The expression distribution and functional mechanisms of DJ‐1 in skeletal muscle are schematically summarized in Figure 1.

### 3.1 Tissue Expression and Subcellular Distribution Mechanisms of DJ‐1 in Skeletal Muscle

Skeletal muscle is among the tissues with high DJ‐1 expression (Mita et al. 2018), consistent with its role as the body's primary metabolic organ. Using two‐dimensional electrophoresis quantitative analysis, researchers found DJ‐1 expression in fast‐twitch fibers (represented by gastrocnemius) was 3.57‐fold higher than in slow‐twitch fibers (represented by soleus) (Roca‐Rivada et al. 2012). This fiber type‐specific expression difference correlates closely with fast‐twitch fibers’ high glycolytic metabolic rates and strong oxidative stress resistance requirements, providing essential clues for understanding DJ‐1's physiological functions.

At the cellular level, DJ‐1 is primarily localized to the cytoplasm under basal conditions (Nagakubo et al. 1997), while its presence is also detected in mitochondria and the nucleus, albeit at relatively lower proportions. Notably, this distribution is not static but dynamically adjusted according to cellular physiological states (Miller et al. 2003; Canet‐Avilés et al. 2004). Li Zhang et al. elucidated the precise localization of DJ‐1 within mitochondria using subcellular fractionation and immunogold electron microscopy techniques: endogenous DJ‐1 specifically distributes to the mitochondrial matrix and intermembrane space, rather than the outer or inner membranes, and this localization remains unaffected by pathological mutations (Zhang et al. 2005).

Post‐translational modifications precisely regulate DJ‐1's subcellular localization. Jun Fan et al. discovered SUMOylation modification as a key regulatory mechanism for nuclear localization. Although endogenous DJ‐1 can be detected in the nucleus, when the K130 site (SUMOylation site) is mutated to arginine, the DJ‐1(K130R) mutant loses nuclear translocation ability and remains mainly in the cytoplasm (Fan et al. 2008). This indicates that SUMOylation modification is a molecular switch that determines DJ‐1's dynamic distribution between the nucleus and cytoplasm, conferring flexibility to execute specific functions in different subcellular compartments according to cellular needs.

Animal model studies further validated DJ‐1's critical functions in skeletal muscle. DJ‐1 knockout mice show progressive motor dysfunction during development, including gait coordination abnormalities, persistent forelimb grip strength decline, and significant locomotor activity reduction (Chandran et al. 2008). Single muscle fiber calcium imaging experiments demonstrated DJ‐1's participation in fine regulation of skeletal muscle contractile function through intracellular Ca^2+^ homeostasis control (Shtifman et al. 2011). Studies in Callipyge sheep (a model of postpartum muscle hypertrophy) and knockout mice further reveal that DJ‐1 plays a central regulatory role in skeletal muscle growth and development by positively regulating the insulin‐like growth factor 1 (IGF‐1) signaling pathway, thereby promoting myofibrillin synthesis and accumulation (Yu et al. 2014).

### 3.2 DJ‐1 Regulation of Mitochondrial Homeostasis

Mitochondrial homeostasis is crucial for skeletal muscle function, with DJ‐1 playing an indispensable role in this process. Growing evidence indicates that DJ‐1 ensures muscle cell energy supply and metabolic stability through precise regulation of mitochondrial structure, function, and dynamic balance.

#### 3.2.1 Maintenance of Mitochondrial Structure and Function

Zhang et al. found that skeletal muscle‐specific DJ‐1 knockout (MDKO) mice exhibited reduced exercise endurance, including significantly diminished performance in grip strength, weight‐bearing, treadmill tests, and exercise intolerance. Mitochondrial function assays revealed that DJ‐1 deficiency markedly decreased respiratory rates dependent on Complex I and Complex II, indicating substantial impairment of oxidative phosphorylation.

Electron microscopy revealed that DJ‐1‐deficient mice exhibited significant ultrastructural abnormalities in skeletal muscle, including enlarged mitochondria and loss of cristae, which directly impaired energy conversion efficiency (Zhang et al. 2023). Mechanistically, DJ‐1 exerts its protective effects not by directly acting on the respiratory chain but by regulating the AKT–FoxO1 signaling pathway.

DJ‐1 deficiency reduces AKT phosphorylation levels, leading to nuclear accumulation and activation of FoxO1, which activates atrophy‐related genes including Tripartite motif‐containing 63/Muscle RING‐finger protein‐1 (TRIM63/MuRF‐1) and F‐box protein 32/Muscle atrophy F‐box protein (FBXO32/Atrogin‐1), thereby promoting protein degradation and accompanied by downregulation of Complex I/II subunits, ultimately triggering mitochondrial dysfunction (Zhang et al. 2023). Notably, despite impaired mitochondrial function, no significant increase in ROS levels was observed under certain experimental conditions. This suggests that DJ‐1's critical role lies more in maintaining metabolic and signaling homeostasis than solely in antioxidant function.

The protective role of DJ‐1 in mitochondrial function is particularly pronounced under stress conditions. McCoy et al. found that DJ‐1 overexpression failed to rescue mitochondrial fragmentation caused by PTEN‐induced kinase 1 (PINK1)/Parkinson disease protein 2 (*parkin*) gene deletion, yet protected PINK1‐deficient cells from rotenone‐induced mitochondrial damage (McCoy and Cookson 2011). Research by Nottia et al. demonstrated that DJ‐1‐deficient fibroblasts exhibited a significant 53% reduction in ATP synthesis capacity when utilizing mitochondrial complex I substrates as an energy source. Under basal conditions, ROS levels in these cells were already markedly elevated, exceeding those of normal cells by 64%; under H_2_O_2_‐induced oxidative stress, ROS levels increased significantly. BNGE and Western blot analyses revealed that DJ‐1 deficiency resulted in a 56% reduction in total mitochondrial complex I, accompanied by decreased expression of multiple subunits (NDUFS1, NDUFA9, NDUFB11, NDUFB4) (Di Nottia et al. 2017).

#### 3.2.2 Regulation of Mitochondrial Dynamic Balance

Mitochondrial dynamics, including fusion and fission, are crucial for maintaining mitochondrial network structure and function (Archer 2013). DJ‐1 maintains mitochondrial dynamic balance through regulating key fusion and fission proteins (Irrcher et al. 2010; Wang et al. 2012).

In mitochondrial fusion, DJ‐1 deficiency leads to downregulation of mitofusin 1 (MFN1) (Irrcher et al. 2010). Irrcher et al. observed significantly fragmented mitochondrial morphology in primary cortical neurons and embryonic fibroblasts from DJ‐1 knockout mice and in lymphoblasts derived from Parkinson's disease patients harboring DJ‐1 mutations. This morphological abnormality directly correlates with reduced MFN1 expression levels, whose downregulation disrupts mitochondrial homeostasis and increases cellular susceptibility to oxidative stress (Irrcher et al. 2010).

Regarding mitochondrial fission regulation, research by Wang et al. indicates that DJ‐1 can influence mitochondrial fission equilibrium by modulating the expression levels of dynamin‐related protein 1 (Drp1). Drp1 levels decreased in wild‐type DJ‐1 overexpressing cells and increased in mutant DJ‐1 cells. Suppressing Drp1 expression via RNA interference corrected mitochondrial morphological abnormalities in mutant DJ‐1 cells and partially improved mitochondrial dysfunction (Wang et al. 2012). Additional studies confirmed that DJ‐1 deficiency causes decreased mitochondrial membrane potential, reduced ATP production, and morphological abnormalities (Hao et al. 2010; Zhou et al. 2017).

While key evidence for DJ‐1 regulating mitochondrial dynamics primarily comes from nervous system studies, recent skeletal muscle research has shown DJ‐1 deficiency causes mitochondrial dysfunction (such as decreased oxidative phosphorylation efficiency, increased ROS production, and reduced transmembrane potential), upregulation of muscle atrophy genes (Zhang et al. 2023; Shi et al. 2015), and possible regulation of mitochondrial dynamics regulatory factors (such as DJ‐1‐miR‐181a axis correlation in aging muscle) (Goljanek‐Whysall et al. 2020). Although these data support extending the concept of DJ‐1 regulating mitochondrial dynamics from the nervous system to skeletal muscle, no studies to date have directly observed mitochondrial fusion/fission abnormalities in primary myotubes or muscle‐specific DJ‐1 knockout mice. Furthermore, the expression patterns and regulatory relationships of MFN1/DRP1 in skeletal muscle DJ‐1 knockout models remain unclear, limiting definitive conclusions regarding DJ‐1's role in muscle mitochondrial dynamics.

Future studies should validate DJ‐1's role in skeletal muscle mitochondrial dynamics through systematic approaches. First, in DJ‐1 knockout or muscle‐specific knockout mice and primary myotube models, mitochondrial fusion/fission alterations should be assessed by combining live‐cell microscopy with MFN1/DRP1 protein expression analysis. Second, causal relationships can be established through rescue experiments: DRP1 inhibition (e.g., using Mdivi‐1 or genetic knockdown) or MFN1 overexpression in DJ‐1‐deficient muscle cells should be evaluated for their ability to restore mitochondrial morphology, respiratory function, and myofiber viability. Third, mechanistic insights can be gained through immunoprecipitation, chromatin immunoprecipitation (ChIP), or miRNA interference to elucidate whether DJ‐1 directly regulates MFN1/DRP1 expression or their upstream signaling pathways. Finally, translational potential can be assessed by testing targeted DRP1 inhibitors or MFN1 activators in sarcopenia or neuromuscular disease models. These systematic investigations will clarify DJ‐1's functional positioning as a central node in skeletal muscle mitochondrial homeostasis and inform therapeutic strategy development.

### 3.3 DJ‐1‐Mediated Oxidative‐Reductive and Metabolic Regulation

As a highly metabolically active tissue, skeletal muscle faces oxidative stress challenges (Sachdev and Davies 2008). Multiple in vitro and in vivo studies demonstrate significant causal relationships between ROS accumulation and sustained muscle contractile capacity decline, confirming oxidative stress's key role in muscle fatigue mechanisms (Powers and Jackson 2008).

#### 3.3.1 DJ‐1‐Mediated Oxidative‐Reductive Balance Regulation

The core of DJ‐1's antioxidant function lies in its conserved cysteine residue Cys‐106. Cys‐106 is first oxidized to sulfinic acid (Cys‐106‐SO_2_H) when intracellular ROS levels increase, triggering DJ‐1 conformational changes that activate its antioxidant functions (Raninga et al. 2017).

DJ‐1 exerts its antioxidant effects through two primary pathways: First, DJ‐1 directly scavenges ROS molecules (represented by H_2_O_2_) via its own oxidation (Taira et al. 2004). Taira et al. Experiments across multiple cell types confirmed that DJ‐1 directly binds to and scavenges H_2_O_2_ through a dimer‐structure‐dependent auto‐oxidation reaction (without requiring other molecules). Mutations (such as the PD‐associated L166P or dimer‐disrupting V51A/C53A) abolish H_2_O_2_ scavenging capacity, making cells sensitive to oxidative stress and leading to cell death (Taira et al. 2004).

DJ‐1 exerts broader protective effects by activating the endogenous antioxidant defense system. In H157 non‐small cell lung cancer cells, siRNA‐mediated silencing of DJ‐1 resulted in an 80% downregulation of NAD(P)H: quinone oxidoreductase 1 (NQO1) mRNA levels, with no significant changes in Nrf2 transcription, suggesting its primary action targets protein stability. ARE‐luciferase reporter assays demonstrated that DJ‐1 deficiency in Huh7 hepatocellular carcinoma cells markedly reduced basal and tert‐butylhydroquinone (tBHQ)‐induced antioxidant response element (ARE) transcriptional activity; in DJ‐1^−^/^−^ mouse embryonic fibroblasts, tBHQ failed to effectively induce the expression of Nrf2 target genes such as NQO1 and Glutamate‐cysteine ligase modifier subunit (GCLM), with reductions of approximately 4.3‐ and 4.26‐fold, respectively.

Further analysis revealed that DJ‐1 deficiency significantly shortens the half‐life of Nrf2 and enhances its ubiquitination levels. Immunoprecipitation experiments confirmed that DJ‐1 reduces the interaction between Nrf2 and Kelch‐like ECH‐associated protein 1 (Keap1), inhibiting Cullin‐3‐dependent ubiquitin‐proteasome degradation (Clements et al. 2006). As the body's primary metabolic and motor organ, skeletal muscle represents a high‐risk tissue for ROS generation. Under high‐intensity exercise, aging, or metabolic stress conditions, mitochondria and NADPH oxidase produce large amounts of ROS, which can easily cause protein and mitochondrial damage without adequate clearance (Zhou et al. 2024).

DJ‐1's direct ROS scavenging action can rapidly reduce H_2_O_2_ levels during acute exercise or oxidative stress, preventing ROS burst damage to myofibers; through Nrf2 stabilization and sustained antioxidant enzyme expression maintenance, DJ‐1 provides long‐term protective barriers for skeletal muscle. Studies in mouse myoblast C2C12 cells confirmed that DJ‐1 regulates high‐fat diet (HFD)–induced ROS production, with its deficiency causing elevated overall ROS levels in skeletal muscle, indicating a direct contribution to muscle cellular environmental homeostasis (Shi et al. 2015).

#### 3.3.2 Oxidative Stress‐Mediated Metabolic Reprogramming

DJ‐1 maintains metabolic homeostasis by regulating skeletal muscle metabolic reprogramming under oxidative stress conditions. It indirectly regulates lipid allocation and glucose balance by reshaping glycolytic flux direction and energy utilization patterns (Shi et al. 2015; Eberhard and Lammert 2017).

DJ‐1‐mediated metabolic regulation exhibits high specificity. Shi et al. reported that in mouse C2C12 myoblasts, DJ‐1 deficiency leads to ROS accumulation and activates AMP‐activated protein kinase (AMPK), upregulating hexokinase 2 (HK2), phosphofructokinase (PFK), and lactate dehydrogenase A (LDHA), thereby inducing a shift in myofibrillar type from oxidative type I to glycolytic type IIa/IIx. Concurrently, ROS signaling promoted Uncoupling Protein 3 (UCP3) upregulation and proton leakage, establishing a “Warburg‐like” metabolic pattern. Although this metabolic reprogramming reduces ATP efficiency, it alleviates ROS overproduction by relieving the electron transport chain load. This reflects an adaptive protective mechanism of cells under oxidative stress. Furthermore, AMPK activation promotes the membrane translocation of Glucose Transporter Type 4 (GLUT4), enhancing glucose uptake and providing ample substrates for glycolysis (Shi et al. 2015).

More importantly, local metabolic remodeling in skeletal muscle affects whole‐body lipid storage through “muscle‐adipose” metabolic crosstalk. DJ‐1 knockout mice under HFD conditions exhibit unique metabolic phenotypes: despite elevated skeletal muscle ROS levels, they show reduced peripheral fat depots, decreased adipocyte volume, reduced circulating leptin and resistin concentrations, while lipogenesis genes and hepatic lipid deposition show no significant changes. This suggests DJ‐1 primarily regulates whole‐body lipid distribution through skeletal muscle metabolic reprogramming rather than directly acting on adipose tissue or liver, highlighting skeletal muscle's vital role as a whole‐body metabolic regulator (Shi et al. 2015).

Notably, DJ‐1 deficiency‐induced metabolic reprogramming exhibits significant environmental dependence and biphasic characteristics. Under normal nutritional conditions, DJ‐1 deficiency does not markedly affect skeletal muscle energy metabolism or insulin sensitivity. However, in chronic nutritional overload scenarios such as high‐fat diets, elevated ROS levels in skeletal muscle become a key triggering factor: DJ‐1 deficiency amplifies ROS accumulation without causing marked oxidative damage, instead triggering AMPK activation, mild mitochondrial uncoupling, and glycolytic pathway reprogramming (Shi et al. 2015).

The physiological significance of this metabolic reprogramming displays marked time‐dependency. During acute oxidative stress (e.g., brief exercise, acute ischemia–reperfusion), the enhanced glycolysis and UCP3‐mediated mild proton leakage induced by DJ‐1 deficiency may serve as a rapid response strategy, limiting damage propagation by reducing mitochondrial ROS generation and exhibiting certain adaptive protective significance. However, under chronic aging‐related oxidative stress, age‐dependent decline in DJ‐1 expression perpetually activates the ROS–AMPK axis, transforming the initially compensatory metabolic reprogramming into a pathological state. Persistent glycolytic bias, reduced proportion of oxidative muscle fibers, weakened mitochondrial function, and AMPK–mTOR signaling imbalance collectively lead to diminished muscle synthetic capacity and muscle fiber atrophy. Both clinical and animal studies indicate that DJ‐1 levels positively correlate with muscle mass, endurance, and mitochondrial health (Zhang et al. 2023), suggesting that DJ‐1 deficiency may be a key driver of metabolic imbalance in age‐related sarcopenia.

While Shi et al.'s discovery of the “UCP3‐AMPK‐GLUT4” axis provides essential insights, its effectiveness is limited by multiple factors, including ROS dosage, UCP3 functional status, AMPK activation thresholds, insulin signaling integrity, and whole‐body metabolic interactions. Additionally, their whole‐body DJ‐1 KO model (non‐muscle conditional knockout) makes it difficult to exclude contributions from other tissues, like adipose tissue or liver. Therefore, future research requires combining muscle‐specific knockout, ROS subcellular localization, and AMPK pathway blockade strategies to clarify whether DJ‐1 represents a viable target for metabolic disease intervention.

### 3.4 DJ‐1‐Mediated Muscle Aging

With aging, DJ‐1 expression in skeletal muscle significantly decreases and positively correlates with muscle mass and function. Aged mice (24 months) showed approximately 30% lower skeletal muscle DJ‐1 protein levels compared to young mice (7 months) (Zhang et al. 2023). More importantly, expression decreases, and oxidized DJ‐1 (oxDJ‐1) accumulation markedly increases. Yuichiro Mita et al. found that oxDJ‐1 levels in skeletal muscle were significantly higher in 130‐week‐old mice compared to 9‐week‐old young mice. Since oxDJ‐1 represents the hyperoxidized form of the DJ‐1 active site, this indicates impaired antioxidant function of DJ‐1 (Mita et al. 2018).

Studies further confirmed DJ‐1's association with muscle health. Age‐related studies showed significantly lower DJ‐1 protein levels in elderly skeletal muscle compared to young adults. Transcriptome data analysis (GSE25941) similarly confirmed DJ‐1 mRNA expression downregulation in elderly populations, suggesting DJ‐1 expression reduction may be an important factor in aging‐related muscle degeneration. Notably, rehabilitation intervention studies provided evidence of DJ‐1 plasticity. In patients undergoing rehabilitation training after cast immobilization, skeletal muscle DJ‐1 expression showed upward trends, indicating DJ‐1's stress response capacity and possible participation in muscle repair processes after injury (Zhang et al. 2023). This finding suggests that regulating DJ‐1 expression may represent a potential strategy for promoting muscle recovery.

Genetic studies have further revealed the critical role of DJ‐1 in maintaining muscle homeostasis. MDKO mice exhibit reduced lean body mass and a 20% decrease in muscle cross‐sectional area as early as young adulthood (5 months) (Zhang et al. 2023). Zebrafish models showed that DJ‐1 knockout individuals exhibited swimming capacity decline and growth stagnation starting at 6–9 months, with significant slow and fast muscle cross‐sectional area reduction and mitochondrial degeneration in old age (2 years) (Rostad et al. 2024).

At the mechanistic level, zebrafish studies reveal that alpha‐actinin‐2 (ACTN2) expression is downregulated to approximately 10% of the wild‐type level, leading to a marked decrease in mitochondrial complex I activity. Concurrently, multiple energy metabolism‐related proteins (such as 2,4‐dienoyl‐CoA reductase 1 (DECR1) and mitochondrial ribosomal large subunit proteins MRPL49, MRPL4, and MRPL30) are markedly reduced, impairing mitochondrial β‐oxidation and self‐maintenance capacity. Correspondingly, muscle aging‐associated metabolic enzymes (Prostaglandin reductase 2 (PTGR2) and glycerol‐3‐phosphate dehydrogenase 1B (GPD1B)) also decrease significantly, accelerating the emergence of aging signals (Rostad et al. 2024).

Furthermore, the role of non‐coding RNAs in DJ‐1 regulation of muscle aging has garnered increasing attention. Li et al. discovered that microRNA‐181a (miR‐181a) can target and regulate DJ‐1 and its associated molecules, such as PRKN/PARK2 (Parkin) and Sequestosome‐1/p62 (SQSTM1/p62). In aged muscle, reduced miR‐181a expression leads to abnormal DJ‐1 expression. By injecting miR‐181a mimics in vivo, researchers successfully restored DJ‐1 expression levels and mitochondrial function in the skeletal muscle of aged mice, improving muscle strength and metabolic status (Goljanek‐Whysall et al. 2020).

Thus, during aging, both DJ‐1 expression decline and abnormal oxidative modification weaken its antioxidant and metabolic protective effects, triggering muscle mass reduction and functional decline. DJ‐1 deficiency model phenotypes reinforce its key status in maintaining skeletal muscle proteostasis and mitochondrial function; simultaneously, upstream regulatory mechanisms like miRNAs provide new research directions for DJ‐1‐targeted anti‐aging interventions.

In summary, DJ‐1 exhibits high subcellular localization dynamics, flexibly translocating between cytoplasm, mitochondria, and nucleus according to cellular stress states, with its predominant expression in fast‐twitch fibers suggesting memorable roles in responding to acute oxidative stress. Functionally, DJ‐1 constructs multi‐layered cellular protection networks: ensuring energy metabolic stability through maintaining mitochondrial structural function and regulating fusion/fission balance; providing comprehensive oxidative stress protection through direct ROS scavenging, Nrf2/ARE pathway activation, and metabolic reprogramming mediation; maintaining muscle tissue integrity through delaying aging‐related functional decline.

### 3.5 Controversies in DJ‐1 Functional Mechanisms and Research Prospects

Although the aforementioned research has revealed DJ‐1's important protective roles in skeletal muscle, some controversies and paradoxes regarding its precise functions require objective discussion. These inconsistent viewpoints mainly focus on the following aspects, and an in‐depth analysis of these controversies helps guide future research directions and provides foundations for understanding disease mechanisms in Chapter 3.

#### 3.5.1 The Paradox of Fast Muscle Predominant Expression and Slow Muscle Protection

Although research indicates DJ‐1 expression in fast‐twitch (type II) fibers is 3.57‐fold higher than in slow‐twitch (type I) fibers (Roca‐Rivada et al. 2012), confusingly, multiple studies find that DJ‐1's protective effects seem more significant in slow muscle. For example, in DJ‐1 knockout mice and zebrafish models, oxidative muscle fibers showed more severe atrophy and degenerative changes than glycolytic muscle fibers (Zhang et al. 2023; Rostad et al. 2024). This contradictory phenomenon of “high expression‐low protection” versus “low expression‐high dependence” may reflect fiber type‐specific metabolic demand differences: fast muscle dependence on rapid energy mobilization requires higher DJ‐1 levels to respond to acute oxidative pressure and glycolytic metabolism byproducts. In contrast, slow muscle forms stronger dependence on DJ‐1's chronic protective functions due to long‐term oxidative metabolic load and sustained mitochondrial activity, making it more susceptible to irreversible damage when deficient.

#### 3.5.2 Cell‐Specific Antioxidant Effects and Functional Redundancy

Controversies also exist regarding DJ‐1's antioxidant function and cell‐specificity. While most studies confirm that DJ‐1 can scavenge H_2_O_2_ and protect cells from oxidative damage (Taira et al. 2004), some studies report that in specific cell types (such as primary cardiomyocytes and some neuronal subtypes), DJ‐1 knockdown does not significantly alter basal ROS levels or oxidative stress sensitivity (Yamaguchi and Shen 2007; Dongworth et al. 2014). This seemingly contradictory phenomenon may reflect different antioxidant redundancy mechanisms across cell types: in tissues highly dependent on oxidative metabolism, multiple parallel antioxidant systems may exist, with one system's deficiency being partially compensated by others. DJ‐1's antioxidant effects may also be stress‐induced, only significantly activated under specific oxidative challenge conditions.

Controversies regarding DJ‐1 functional redundancy and compensatory mechanisms primarily stem from inconsistent phenotypes in whole‐body knockout mouse studies. Initial DJ‐1 knockout mouse studies reported relatively mild phenotypes (Chandran et al. 2008), while subsequent studies reported more extensive metabolic, cardiovascular, and muscle abnormalities (Zhang et al. 2023; Shi et al. 2015; Wang et al. 2021). These differences may stem from compensatory mechanism variations under different experimental conditions. One possible explanation is that DJ‐1 functional deficiency triggers compensatory upregulation of other protective pathways (such as direct Nrf2 activation, PINK1/Parkin pathway enhancement) (Clements et al. 2006; Imberechts et al. 2022), partially masking deficiency phenotypes. This compensation has temporal and tissue specificity: it is more effective during early development or mild stress, while compensation capacity declines during aging or chronic stress conditions.

Despite the above controversies, these findings establish DJ‐1's core status as a skeletal muscle cytoprotective factor overall. More importantly, these functional controversies provide essential clues for understanding DJ‐1's role variations under different pathological states, particularly its differential manifestations in muscle atrophy, neuromuscular diseases, and metabolic myopathies, which will be further addressed in the next chapter's discussion of skeletal muscle diseases.

## 4 DJ‐1 and Skeletal Muscle‐Related Diseases

**FIGURE 2:**
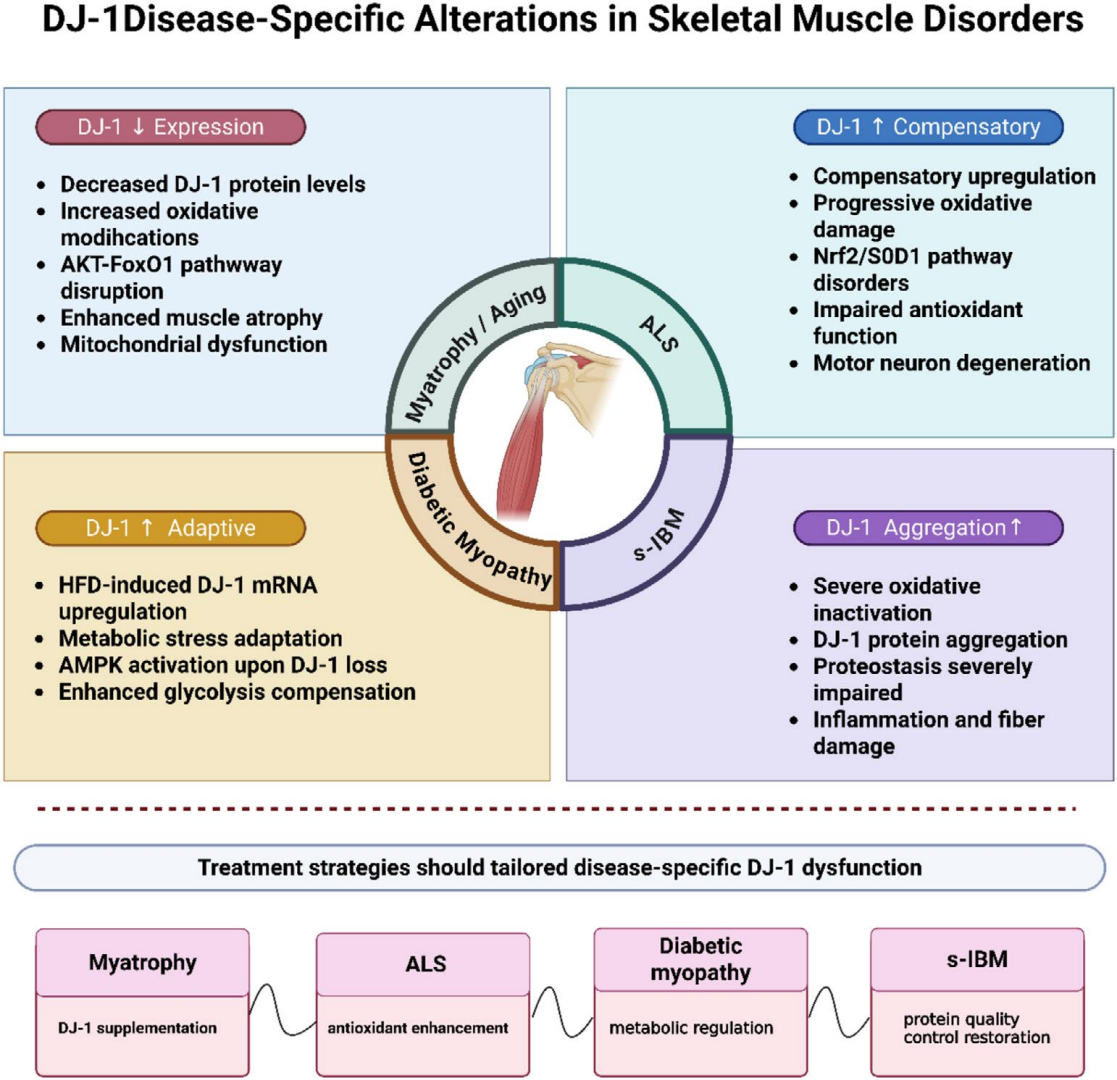
Disease‐specific DJ‐1 alterations in skeletal muscle disorders. DJ‐1 exhibits distinct pathological patterns across major muscle diseases. In myatrophy/aging, DJ‐1 expression decreases with increased oxidative modifications, leading to AKT‐FoxO1 pathway disruption. In ALS, DJ‐1 shows compensatory upregulation but suffers progressive oxidative damage with Nrf2/SOD1 pathway disorders. In diabetic myopathy, a high‐fat diet induces adaptive DJ‐1 mRNA upregulation as a metabolic stress response; DJ‐1 deficiency leads to AMPK activation and enhanced glycolysis. In s‐IBM, severe DJ‐1 aggregation occurs with oxidative inactivation, compromising proteostasis. These disease‐specific patterns indicate that therapeutic approaches should be tailored accordingly: DJ‐1 supplementation for myatrophy, antioxidant enhancement for ALS, metabolic regulation for diabetic myopathy, and protein quality control restoration for s‐IBM. Created in https://BioRender.com.

The involvement of DJ‐1 in skeletal muscle–related diseases and its potential therapeutic implications are summarized in Figure 2.

### 4.1 DJ‐1 and Muscle Atrophy

Muscle atrophy is a common outcome of multiple pathological states involving complex etiologies, including disuse, aging, and cachexia. DJ‐1, a key cytoprotective factor, is closely associated with muscle atrophy development and progression through its expression deficiency or functional disorders.

Knockout mouse models first confirmed DJ‐1's role in maintaining muscle mass. A 2008 pioneering study found that DJ‐1 whole‐body knockout mice exhibited obvious motor dysfunction, including reduced activity, gait abnormalities, and decreased forelimb grip strength (Chandran et al. 2008). Subsequent studies further revealed these mice had reduced lean body mass and decreased motor activity, suggesting DJ‐1 deficiency's widespread effects on the muscular system (Seyfarth et al. 2015). The establishment of the MDKO mouse model in 2023 marks a significant breakthrough in research. Unlike whole‐body knockout, MDKO models exclude interference from other tissues and directly demonstrate DJ‐1's autonomous role in skeletal muscle. MDKO mice showed typical muscle atrophy phenotypes even under normal diet conditions: significantly reduced myofiber cross‐sectional area, decreased muscle contractile strength, and reduced exercise endurance. Histological analysis revealed abnormal mitochondrial structure and impaired oxidative phosphorylation capacity, providing direct evidence for DJ‐1's muscle protective mechanisms (Zhang et al. 2023).

Zebrafish models provide evolutionary perspectives for DJ‐1 function research. DJ‐1 knockout zebrafish exhibited significant muscle atrophy, particularly a 2.2‐fold reduction in red muscle fiber cross‐sectional area and a 1.4‐fold reduction in white muscle fiber area. Accompanying molecular alterations include decreased Nicotinamide Adenine Dinucleotide (NAD^+^/NADH) ratio and enhanced inflammatory responses, suggesting that DJ‐1 deficiency triggers metabolic dysregulation and an inflammatory cascade (Zhou et al. 2024). The high conservation between zebrafish and humans in muscle structure and molecular pathways makes these findings important for understanding human muscle diseases (Daya et al. 2020).

DJ‐1 regulates muscle mass through multiple mechanisms, with the most critical being regulation of the ubiquitin‐proteasome system (UPS). Kim et al., using malnutrition‐induced muscle atrophy models, systematically evaluated DJ‐1's regulatory mechanisms in skeletal muscle atrophy processes. Their model showed that DJ‐1 deficiency led to significant upregulation of TRIM63/MuRF‐1 and FBXO32/Atrogin‐1 expression, accelerating muscle protein degradation. Conversely, DJ‐1 overexpression effectively inhibited these atrophic gene expressions, maintaining protein homeostasis. Oxidative stress signal regulation represents the core mechanism of DJ‐1's protective effects. In the L6 myoblast nutrient deprivation model, DJ‐1 blocks downstream atrophy signaling by inhibiting ROS‐mediated activation of p38 mitogen‐activated protein kinase (p38 MAPK) and stress‐activated protein kinase/c‐Jun N‐terminal kinase (SAPK/JNK). After 24 h of nutrient deprivation, wild‐type cells showed 32.2% increased total DJ‐1 and 25.2% increased oxidized DJ‐1, demonstrating dynamic responses as oxidative stress sensors. DJ‐1 knockdown exacerbated p38/JNK phosphorylation and atrophy marker expression, while overexpression significantly reversed these changes (Kim et al. 2015).

Combined with Zhang et al. (2023) MDKO mouse studies, DJ‐1's UPS regulation occurs not through direct action but through upstream regulation via FoxO1 transcription factors: decreased DJ‐1 expression levels reduce AKT activity, causing dephosphorylated FoxO1 to enter the nucleus and directly bind TRIM63 and FBXO32 promoter regions, driving their transcriptional activation and ultimately activating UPS pathways (Zhang et al. 2023).

Maintaining mitochondrial function is another critical aspect of DJ‐1's anti‐atrophy effects. DJ‐1 deficiency leads to dysfunction of mitochondrial respiratory chain complexes, creating a vicious cycle that promotes atrophy. In MDKO mice, skeletal muscle mitochondria exhibit abnormal morphology and disrupted cristae architecture, with markedly reduced oxidative phosphorylation efficiency. These mitochondrial defects compromise energy supply and accelerate muscle loss by activating mitochondria‐dependent atrophy signaling pathways (Zhang et al. 2023).

Notably, DJ‐1's protective effects show dose and time dependence. Moderate upregulation (such as exercise‐induced) effectively counters atrophy, but under severe stress or late disease stages, endogenous DJ‐1 upregulation may be insufficient to reverse pathological processes. This suggests that enhancing DJ‐1 function during therapeutic windows may have better intervention effects.

### 4.2 DJ‐1 and Amyotrophic Lateral Sclerosis (ALS)

Amyotrophic lateral sclerosis (ALS) is a fatal neurodegenerative disease characterized by progressive motor neuron loss. DJ‐1, as an oxidative stress sensor, plays key regulatory roles in ALS neuro‐muscular pathological cascades.

Importantly, pathological changes in the superoxide dismutase 1 (SOD1) protein pathology exist in all ALS subtypes—Trist et al. found that regardless of SOD1 mutation status, ALS patients exhibit SOD1 protein structural disorders and metal imbalances (Trist et al. 2022). Lev et al., using SOD1(G93A) transgenic mice, found significantly upregulated DJ‐1 expression in the brain and spinal cord with disease progression, accompanied by increased oxidative modification forms (Lev et al. 2009).

The SOD1G93A/DJ‐1−/− double knockout mouse model, constructed by Lev's team, systematically validated DJ‐1's protective effects. DJ‐1 deficiency significantly accelerated ALS disease progression: double knockout mice showed markedly advanced disease onset times, with females advanced by 29 days (Day 83 vs. 112) and males by 12 days (Day 96 vs. 108). Motor function decline accelerated, with females showing motor deficits 25 days earlier (Day 97 vs. 122) and males 28 days earlier (Day 94 vs. 122). Survival analysis showed double knockout mice had 18.4 days shortened average survival (143.4 vs. 161.8 days) (Lev et al. 2015).

These studies confirm DJ‐1 as a disease modifier, comprehensively accelerating ALS progression, revealing that DJ‐1 functional status may determine disease progression speed and severity. Although not a direct pathogenic factor, DJ‐1 dysfunction significantly reduces motor neuron resistance to multiple injuries, including oxidative stress and excitotoxicity. This suggests maintaining or enhancing DJ‐1 function may represent critical therapeutic strategies for delaying ALS progression.

DJ‐1 deficiency‐induced ALS phenotype exacerbation stems from its multi‐level protective mechanisms:

**Oxidative Stress Defense:** Oxidative stress plays key roles in ALS occurrence and progression (Park and Yang 2021), with DJ‐1 resisting this damage through multiple pathways. Research has found that DJ‐1 promotes the nuclear translocation of Extracellular Signal‐Regulated Kinase 1/2 (ERK1/2) and phosphorylates ETS‐like Transcription Factor 1 (Elk1), increasing SOD1 expression to enhance antioxidant capacity (Wang et al. 2011). Simultaneously, DJ‐1 promotes Keap1‐Nrf2 dissociation, enabling Nrf2 nuclear entry to activate NQO1 and other antioxidant enzyme gene expression (Clements et al. 2006; Moscovitz et al. 2015; Yan et al. 2015), which is vital for resisting Nrf2 pathway dysfunction observed in ALS patients and SOD1G93A cell models (Sarlette et al. 2008; Wang et al. 2014; Bono et al. 2021).

**Mitochondrial Homeostasis Maintenance:** ALS patients' spinal motor neurons and muscle tissues commonly exhibit mitochondrial morphological damage, reduced calcium buffering capacity, and respiratory chain dysfunction (Zhao et al. 2022). DJ‐1 effectively alleviates these abnormalities by stabilizing mitochondrial membrane potential (Giaime et al. 2012), promoting Pink1/Parkin‐mediated mitochondrial quality control (Imberechts et al. 2022), and directly scavenging mitochondrial ROS (Xu et al. 2018).

**Metabolic Adaptation Regulation:** ALS patients often show weight loss and energy homeostasis imbalances before onset (Guillot et al. 2021), with motor neurons susceptible to energy metabolic abnormalities due to high ATP demands (Soares et al. 2023). DJ‐1 maintains the mitochondrial localization of HK1 (Hauser et al. 2017) and balances glycolytic flux (Solana‐Manrique et al. 2020), potentially valuable for resisting glucose metabolism decline and lipid metabolism compensation observed in ALS patient cells (Szelechowski et al. 2018). The DJ‐1 defect model also demonstrates reduced branched‐chain amino acids (BCAAs) (Solana‐Manrique et al. 2022), possibly related to protein synthesis disorders in ALS patient muscle.

**Vascular‐Neural Unit Regulation:** Vascular dysfunction is increasingly recognized as an essential factor in ALS neuronal degeneration. Clinical studies have found that levels of Vascular Endothelial Growth Factor (VEGF) in the cerebrospinal fluid of early‐stage ALS patients are significantly reduced (Devos et al. 2004), failing to upregulate normally even under hypoxic conditions, showing “paradoxical responses” (Just et al. 2007). Monocytes from ALS patients exhibit insufficient VEGF production under hypoxic conditions, accompanied by excessive activation of Prolyl Hydroxylase Domain‐2 (PHD‐2) (Moreau et al. 2011). DJ‐1 exerts protective effects in ischemic models by stabilizing Hypoxia‐Inducible Factor‐1 alpha (HIF‐1α) (Parsanejad et al. 2014; Zheng et al. 2018), thereby promoting angiogenesis and metabolic adaptation gene expression, exerting protective effects in ischemic models (Zhao et al. 2021; Wang et al. 2022), which is potentially crucial for resisting hypoxic protective mechanism damage in ALS patients.

### 4.3 DJ‐1 and Sporadic Inclusion Body Myositis (s‐IBM)

Sporadic inclusion body myositis (s‐IBM) is the most common acquired myopathy in individuals over 50. It is characterized by progressive muscle weakness, muscle atrophy, and characteristic pathological changes. s‐IBM muscle biopsies present a unique “triad”: inflammatory cell infiltration, autophagic vacuoles (Askanas et al. 2015), and protein aggregates, figuratively called “muscle Alzheimer's disease.” Recent research indicates that DJ‐1 plays multiple regulatory roles in s‐IBM's complex pathological networks.

Terracciano's team systematically analyzed DJ‐1 expression profiles in s‐IBM patient muscle, finding significantly increased DJ‐1 monomers and dimers, with DJ‐1 immunopositive aggregates observed in approximately 15% of diseased muscle fibers. Double‐labeling gold immunoelectron microscopy showed DJ‐1 distributed in spots within diseased muscle fibers, co‐localizing with mitochondrial markers, suggesting participation in mitochondrial protection. Immunoprecipitation and OxyBlot detection confirmed that DJ‐1 in s‐IBM patient muscle exists in highly oxidized states (Terracciano et al. 2008). Although this study did not directly test DJ‐1's antioxidant functional activity, combined with known associations between DJ‐1 oxidative modification and functional loss, this highly oxidized state likely causes impaired antioxidant function. Notably, DJ‐1's “compensatory upregulation but functional exhaustion” pattern in s‐IBM strikingly resembles manifestations in Alzheimer's and Parkinson's diseases, suggesting that neurodegenerative diseases and s‐IBM may share specific basic pathological mechanisms.

Regarding oxidative stress, s‐IBM muscle fibers commonly exhibit oxidative damage and nitrosative stress (Yang et al. 1996), which damages mitochondria and promotes abnormal tubulin‐associated unit (tau) phosphorylation and aggregation. DJ‐1 typically maintains cellular oxidative‐reductive balance through scavenging ROS. However, in s‐IBM, despite elevated DJ‐1 expression levels, it exists in highly oxidized and aggregated forms, causing antioxidant function loss (Terracciano et al. 2008). This “quantitative compensatory elevation but functional inactivation” contradiction prevents muscle fibers from effectively clearing ROS. Mitochondrial pathological abnormalities represent another significant s‐IBM feature, including giant mitochondria, COX‐negative fibers, mitochondrial DNA deletions, and mitochondrial autophagy disorders, ultimately causing ATP synthesis and energy failure (Askanas et al. 2015). DJ‐1 under physiological states can maintain mitochondrial homeostasis by stabilizing mitochondrial membrane potential and regulating respiratory chain complex activity.

Regarding calcium homeostasis, s‐IBM muscle fibers frequently exhibit abnormal sarcoplasmic reticulum (SR) calcium release and elevated resting cytoplasmic calcium concentrations (Shtifman et al. 2010). The MLC‐βAPP transgenic mouse model developed by Alexander Shtifman's team demonstrated significantly reduced Ca^2+^ transient peaks and prolonged decay times (Moussa et al. 2006). Liposome experiments confirmed that Aβ_1‐42_ directly enhances the open probability of Ryanodine Receptor type 1 (RyR1). Skeletal muscle from DJ‐1 knockout mice also exhibited elevated resting [Ca^2+^]i and restricted SR calcium release, while RyR1 and Sarco/Endoplasmic Reticulum Ca^2+^‐ATPase 1 (SERCA1) expression showed no significant changes. Antioxidants (resveratrol, glutathione) partially restored these abnormalities, suggesting DJ‐1 may regulate skeletal muscle calcium homeostasis by alleviating oxidative stress and maintaining SR calcium channel function (Shtifman et al. 2011).

Based on DJ‐1's multiple protective effects, developing small‐molecule DJ‐1 stabilizers to prevent oxidative inactivation or combined antioxidant use to enhance DJ‐1 function should be considered. DJ‐1 levels and oxidative modification degrees in serum or muscle tissue may be biomarkers for evaluating s‐IBM disease progression.

### 4.4 Diabetic Myopathy

Diabetic myopathy is one of the common microvascular complications of diabetes, characterized by reduced muscle strength, decreased endurance, and muscle atrophy (D'Souza et al. 2013). Preliminary studies show significantly elevated oxidative stress levels in skeletal muscle of type 2 diabetes and high‐fat diet models, accompanied by DJ‐1 expression changes. Shi et al. found that HFD can induce DJ‐1 mRNA upregulation in mouse skeletal muscle, suggesting DJ‐1 as an adaptive response molecule to metabolic stress; in DJ‐1‐deficient mice, ROS levels further increase, causing reduced mitochondrial coupling efficiency, AMPK activation, and enhanced glycolysis. Although these mice show increased energy expenditure and protection against obesity and insulin resistance, results emphasize DJ‐1's key regulatory role in maintaining skeletal muscle energy metabolic homeostasis (Shi et al. 2015). Additionally, Daniel Eberhard and Eckhard Lammert's review indicates that under diabetic conditions, DJ‐1 can buffer metabolic pressure through regulating ROS, stabilizing mitochondria, and participating in insulin signaling maintenance (Eberhard and Lammert 2017). These findings suggest that DJ‐1 dysfunction may contribute to the occurrence of diabetic myopathy, providing new perspectives for understanding insulin resistance and muscle metabolic abnormalities.

It should be noted that the aforementioned conclusions are primarily derived from rodent models, while the oxidative modification status of DJ‐1 in human skeletal muscle and its association with muscle dysfunction remain unexplored. To our knowledge, no published studies have directly quantified DJ‐1 oxidation levels in muscle biopsies from patients with type 2 diabetes mellitus (T2DM), representing an important knowledge gap that warrants investigation.

Based on the robust animal experimental evidence, we hypothesize that in T2DM patients, skeletal muscle DJ‐1 may undergo functional inactivation due to chronic oxidative stress, thereby contributing to metabolic dysregulation and functional decline. Future clinical studies could address this gap through the following approach: (1) Quantify DJ‐1 oxidation levels (e.g., sulfonation of Cys106) in muscle biopsy specimens from T2DM patients versus age‐matched healthy controls; (2) Assess correlations with functional parameters including muscle strength (grip strength, chair stand test), exercise capacity (6‐min walk test), and metabolic indices (HbA1c, HOMA‐IR); (3) Perform longitudinal analyses to determine whether DJ‐1 oxidation status predicts disease progression or therapeutic response. Such investigations would not only validate the translational relevance of rodent findings but also establish DJ‐1 as a potential biomarker or therapeutic target in diabetic myopathy, thereby enhancing the clinical impact of this research direction.

## 5 Therapeutic Potential of DJ‐1 as a Target

DJ‐1 possesses unique advantages as a therapeutic target for skeletal muscle diseases: as a multifunctional protective protein, it can simultaneously regulate multiple pathological elements, including oxidative stress, mitochondrial function, and protein homeostasis; although various myopathies show DJ‐1 expression upregulation, functional insufficiency suggests that enhancing its activity has potential therapeutic value. Some small‐molecule compounds have already shown potential for regulating DJ‐1 function in experimental studies. This section evaluates research progress on these compounds.

Due to their high oral bioavailability, low production costs, and easy dose adjustment advantages, small molecule compounds have become important research directions for DJ‐1‐targeted therapy. Current research strategies mainly include direct DJ‐1 activation, regulation of its related signaling networks, and the use of DJ‐1 inhibitors as research tools. Table 1 summarizes the characteristics and mechanisms of major DJ‐1 regulatory compounds.

**TABLE 1:** Characteristics and mechanisms of major DJ‐1 regulatory compounds.

Compound	Type	Mechanism	Experimental model	Key effects	Limitations	References
Resveratrol (RSV)	Polyphenol	Upregulates DJ‐1 expression, promotes mitochondrial localization, and enhances SIRT1 activity.	H9c2 cells, DJ‐1 KO muscle fibers	Restores mitochondrial complex I activity, reduces resting calcium levels by 51%	Non‐specific targets, low bioavailability	(Shtifman et al. 2011; Zhang et al. 2018; Howitz et al. 2003)
SRT2104	Synthetic SIRT1 activator	Activates SIRT1‐PGC‐1α axis, reduces PGC‐1α acetylation	mdx mice	30%–50% increased complex activity, 2.1‐fold increase in oxidative muscle fibers	Indirect DJ‐1 association	(Giovarelli et al. 2025)
Compound 23	DJ‐1 inhibitor	Direct interaction with endogenous DJ‐1	Mouse muscle local injection	Regulates AKT‐FoxO1 pathway, 25% reduction in muscle fiber CSA	Research tool, not therapeutic drug	(Zhang et al. 2023)
UCP0054278	DJ‐1 protein‐specific regulator	Direct DJ‐1 binding stabilizes functional conformation	Parkinson's disease cell model	Regulates Nrf2 through PTEN‐PI3K/Akt pathway	Not validated in muscle diseases	(Yanagida et al. 2009; Inden et al. 2011)

Resveratrol (RSV), the most extensively studied DJ‐1 modulator, exhibits multi‐level regulatory effects. In hypoxia/reoxygenation (H/R)‐treated H9c2 cells, RSV upregulates DJ‐1 protein expression and promotes its mitochondrial translocation, restoring mitochondrial complex I activity in a DJ‐1‐dependent manner (Zhang et al. 2018). In DJ‐1 knockout skeletal muscle cells, RSV reduces abnormally elevated resting calcium levels (282.9 nM) by approximately 51% (to 137.4 nM) (Shtifman et al. 2011). Mechanistic studies reveal that RSV acts through DJ‐1 to synergize with the SIRT1‐PGC‐1α signaling axis: DJ‐1 directly enhances the deacetylase activity of Sirtuin 1 (SIRT1), thereby activating PGC‐1α to promote mitochondrial biogenesis and antioxidant gene expression (Takahashi‐Niki et al. 2016).

SRT2104, as a new‐generation SIRT1 activator, is 1000‐fold more active than RSV. In mdx muscular dystrophy mice, SRT2104 significantly reduced PGC‐1α acetylation levels (42% reduction), enhanced mitochondrial respiratory chain complex activity (30%–50% increase), and promoted muscle fiber transformation toward oxidative types (2.1‐fold proportion increase) (Giovarelli et al. 2025). Although SRT2104's association with DJ‐1 is indirect, considering DJ‐1's close interactions with the SIRT1‐PGC‐1α axis, such compounds provide proof‐of‐concept for achieving therapeutic effects through regulating DJ‐1‐related signaling networks.

Compound 23, as a DJ‐1‐specific antagonist, though not a therapeutic drug, has unique value in mechanistic research. Mouse experiments confirmed local injection of compound 23 caused AKT‐FoxO1 signaling pathway inhibition, reducing muscle fiber cross‐sectional area by 25% (Zhang et al. 2023), clarifying DJ‐1‐AKT‐FoxO1's status as a key skeletal muscle metabolic regulatory axis.

Structure‐based drug design has achieved preliminary progress. Small molecules like UCP0054278 can directly bind the DJ‐1 protein, stabilizing its functional conformation and preventing excessive C106 site oxidation (Yanagida et al. 2009; Inden et al. 2011). Although these compounds are mainly validated in Parkinson's disease models, their direct DJ‐1‐targeting strategies provide references for muscle disease therapy.

Although DJ‐1‐targeted therapy research remains early, technological advances bring new opportunities to this field. Combining structural biology with computational drug design may develop more specific DJ‐1 regulators, while nanotechnology will provide more precise muscle‐targeting strategies. Multi‐omics technology applications will help establish comprehensive DJ‐1 functional status assessment systems, laying the foundations for personalized therapy.

More importantly, DJ‐1's synergistic effects with other cytoprotective pathways (PINK1/Parkin, Nrf2/ARE, SIRT1/PGC‐1α, etc.) provide theoretical support for combination therapies. By systematically mapping interaction networks of these pathways, “1 + 1 > 2” therapeutic effects may be achieved. With continued elucidation of DJ‐1 mechanisms and accumulated experience from nervous system disease research, DJ‐1‐targeted therapy may provide new treatment options for refractory muscle diseases like ALS and s‐IBM.

## 6 Conclusions and Prospects

This review systematically elucidates DJ‐1's key roles in skeletal muscle homeostasis maintenance and disease occurrence, providing essential perspectives for understanding muscle disease molecular mechanisms and developing therapeutic strategies.

DJ‐1, an evolutionarily conserved multifunctional regulatory protein, exerts core protective effects in skeletal muscle through its unique oxidative‐reductive sensing‐effector mechanisms. Its predominant expression in fast‐twitch fibers, dynamic subcellular localization, and precise oxidative modification of critical cysteine residues (Cys‐106) enable real‐time cellular oxidative status monitoring and corresponding protective pathway activation. DJ‐1 constructs comprehensive skeletal muscle cellular protection networks through maintaining mitochondrial structural function, regulating oxidative‐reductive balance, mediating metabolic reprogramming, and delaying aging processes via multiple mechanisms.

Regarding disease associations, DJ‐1 dysfunction closely correlates with muscle atrophy, ALS, s‐IBM, and various muscle diseases. Notably, these diseases commonly exhibit “DJ‐1 expression compensatory upregulation but functional impairment” patterns, suggesting DJ‐1 serves as a key regulatory factor in disease progression and potentially as a biomarker for disease severity and prognosis assessment.

Therapeutic target research shows small molecule regulators represented by resveratrol have demonstrated preliminary efficacy in cellular and animal models for regulating DJ‐1 function, providing proof‐of‐concept for drug development. However, DJ‐1‐specific regulators for skeletal muscle diseases remain in early research stages, with most compounds having limitations of target non‐specificity or being limited to in vitro validation.

Future research should focus on: (1) elucidating DJ‐1's specific mechanisms in different muscle fiber types and their relationships with muscle disease heterogeneity; (2) developing high‐specificity, high‐bioavailability DJ‐1 regulators with systematic preclinical evaluation; (3) exploring DJ‐1's synergistic effects with other cytoprotective pathways to provide theoretical foundations for combination therapy strategies; (4) conducting clinical cohort studies to validate DJ‐1's value as a muscle disease diagnostic and prognostic marker.

With a deepening understanding of DJ‐1 molecular mechanisms and advances in targeted drug development technologies, DJ‐1 may become an essential target for precision muscle disease therapy, providing new treatment options for improving patient prognosis and quality of life.

## Funding

This research was supported by the National Natural Science Foundation of China (Grant 12072202).

## Ethics Statement

The authors have nothing to report.

## Consent

The authors have nothing to report.

## Conflicts of Interest

The authors declare no conflicts of interest.

## Data Availability

The authors have nothing to report.

## References

[cph470091-bib-0001] Abu Shelbayeh, O. , T. Arroum , S. Morris , and K. B. Busch . 2023. “PGC‐1α Is a Master Regulator of Mitochondrial Lifecycle and ROS Stress Response.” Antioxidants (Basel) 12, no. 5: 1075. 37237941 10.3390/antiox12051075 PMC10215733

[cph470091-bib-0002] Archer, S. L. 2013. “Mitochondrial Dynamics–Mitochondrial Fission and Fusion in Human Diseases.” New England Journal of Medicine 369, no. 23: 2236–2251. 24304053 10.1056/NEJMra1215233

[cph470091-bib-0003] Askanas, V. , W. K. Engel , and A. Nogalska . 2015. “Sporadic Inclusion‐Body Myositis: A Degenerative Muscle Disease Associated With Aging, Impaired Muscle Protein Homeostasis and Abnormal Mitophagy.” Biochimica et Biophysica Acta 1852, no. 4: 633–643. 25241263 10.1016/j.bbadis.2014.09.005

[cph470091-bib-0004] Bandyopadhyay, S. , and M. R. Cookson . 2004. “Evolutionary and Functional Relationships Within the DJ1 Superfamily.” BMC Evolutionary Biology 4: 6. 15070401 10.1186/1471-2148-4-6 PMC385224

[cph470091-bib-0005] Bauer, J. , J. E. Morley , A. M. W. J. Schols , et al. 2019. “Sarcopenia: A Time for Action. An SCWD Position Paper.” Journal of Cachexia, Sarcopenia and Muscle 10, no. 5: 956–961. 31523937 10.1002/jcsm.12483 PMC6818450

[cph470091-bib-0006] Blackinton, J. , M. Lakshminarasimhan , K. J. Thomas , et al. 2009. “Formation of a Stabilized Cysteine Sulfinic Acid Is Critical for the Mitochondrial Function of the Parkinsonism Protein DJ‐1.” Journal of Biological Chemistry 284, no. 10: 6476–6485. 19124468 10.1074/jbc.M806599200 PMC2649108

[cph470091-bib-0007] Bonifati, V. , P. Rizzu , M. van Baren , et al. 2003. “Mutations in the DJ‐1 Gene Associated With Autosomal Recessive Early‐Onset Parkinsonism.” Science 299, no. 5604: 256–259. 12446870 10.1126/science.1077209

[cph470091-bib-0008] Bono, S. , M. Feligioni , and M. Corbo . 2021. “Impaired Antioxidant KEAP1‐NRF2 System in Amyotrophic Lateral Sclerosis: NRF2 Activation as a Potential Therapeutic Strategy.” Molecular Neurodegeneration 16, no. 1: 71. 34663413 10.1186/s13024-021-00479-8 PMC8521937

[cph470091-bib-0009] Canet‐Avilés, R. M. , M. A. Wilson , D. W. Miller , et al. 2004. “The Parkinson's Disease Protein DJ‐1 Is Neuroprotective due to Cysteine‐Sulfinic Acid‐Driven Mitochondrial Localization.” Proceedings of the National Academy of Sciences of the United States of America 101, no. 24: 9103–9108. 15181200 10.1073/pnas.0402959101 PMC428480

[cph470091-bib-0010] Chandran, J. S. , X. Lin , A. Zapata , et al. 2008. “Progressive Behavioral Deficits in DJ‐1‐Deficient Mice Are Associated With Normal Nigrostriatal Function.” Neurobiology of Disease 29, no. 3: 505–514. 18187333 10.1016/j.nbd.2007.11.011 PMC2271119

[cph470091-bib-0011] Chen, K. , P. Gao , Z. Li , et al. 2022. “Forkhead Box O Signaling Pathway in Skeletal Muscle Atrophy.” American Journal of Pathology 192, no. 12: 1648–1657. 36174679 10.1016/j.ajpath.2022.09.003

[cph470091-bib-0012] Chen, L. K. , J. Woo , P. Assantachai , et al. 2020. “Asian Working Group for Sarcopenia: 2019 Consensus Update on Sarcopenia Diagnosis and Treatment.” Journal of the American Medical Directors Association 21, no. 3: 300–307. 32033882 10.1016/j.jamda.2019.12.012

[cph470091-bib-0013] Clements, C. M. , R. S. McNally , B. J. Conti , T. W. Mak , and J. P. Ting . 2006. “DJ‐1, a Cancer‐ and Parkinson's Disease‐Associated Protein, Stabilizes the Antioxidant Transcriptional Master Regulator Nrf2.” Proceedings of the National Academy of Sciences of the United States of America 103, no. 41: 15091–15096. 17015834 10.1073/pnas.0607260103 PMC1586179

[cph470091-bib-0014] Daya, A. , R. Donaka , and D. Karasik . 2020. “Zebrafish Models of Sarcopenia.” Disease Models & Mechanisms 13, no. 3: dmm042689. 32298234 10.1242/dmm.042689 PMC7132805

[cph470091-bib-0015] Devos, D. , C. Moreau , P. Lassalle , et al. 2004. “Low Levels of the Vascular Endothelial Growth Factor in CSF From Early ALS Patients.” Neurology 62, no. 11: 2127–2129. 15184633 10.1212/01.wnl.0000129913.44351.a3

[cph470091-bib-0016] Di Nottia, M. , M. Masciullo , D. Verrigni , et al. 2017. “DJ‐1 Modulates Mitochondrial Response to Oxidative Stress: Clues From a Novel Diagnosis of PARK7.” Clinical Genetics 92, no. 1: 18–25. 27460976 10.1111/cge.12841

[cph470091-bib-0017] Dolgacheva, L. P. , A. V. Berezhnov , E. I. Fedotova , V. P. Zinchenko , and A. Y. Abramov . 2019. “Role of DJ‐1 in the Mechanism of Pathogenesis of Parkinson's Disease.” Journal of Bioenergetics and Biomembranes 51, no. 3: 175–188. 31054074 10.1007/s10863-019-09798-4 PMC6531411

[cph470091-bib-0018] Dongworth, R. K. , U. A. Mukherjee , A. R. Hall , et al. 2014. “DJ‐1 Protects Against Cell Death Following Acute Cardiac Ischemia‐Reperfusion Injury.” Cell Death & Disease 5, no. 2: e1082. 24577080 10.1038/cddis.2014.41 PMC3944257

[cph470091-bib-0019] D'Souza, D. M. , D. Al‐Sajee , and T. J. Hawke . 2013. “Diabetic Myopathy: Impact of Diabetes Mellitus on Skeletal Muscle Progenitor Cells.” Frontiers in Physiology 4: 379. 24391596 10.3389/fphys.2013.00379 PMC3868943

[cph470091-bib-0020] Eberhard, D. , and E. Lammert . 2017. “The Role of the Antioxidant Protein DJ‐1 in Type 2 Diabetes Mellitus.” Advances in Experimental Medicine and Biology 1037: 173–186. 29147909 10.1007/978-981-10-6583-5_11

[cph470091-bib-0021] Fan, J. , H. Ren , E. Fei , et al. 2008. “Sumoylation Is Critical for DJ‐1 to Repress p53 Transcriptional Activity.” FEBS Letters 582, no. 7: 1151–1156. 18339323 10.1016/j.febslet.2008.03.003

[cph470091-bib-0022] Forman, H. J. , and H. Zhang . 2021. “Targeting Oxidative Stress in Disease: Promise and Limitations of Antioxidant Therapy.” Nature Reviews. Drug Discovery 20, no. 9: 689–709. 34194012 10.1038/s41573-021-00233-1 PMC8243062

[cph470091-bib-0023] Giaime, E. , H. Yamaguchi , C. A. Gautier , T. Kitada , and J. Shen . 2012. “Loss of DJ‐1 Does Not Affect Mitochondrial Respiration but Increases ROS Production and Mitochondrial Permeability Transition Pore Opening.” PLoS One 7, no. 7: e40501. 22792356 10.1371/journal.pone.0040501 PMC3392228

[cph470091-bib-0024] Giovarelli, M. , S. Zecchini , S. R. Casati , et al. 2025. “The SIRT1 Activator SRT2104 Exerts Exercise Mimetic Effects and Promotes Duchenne Muscular Dystrophy Recovery.” Cell Death & Disease 16, no. 1: 259. 40195304 10.1038/s41419-025-07595-z PMC11977210

[cph470091-bib-0025] Goljanek‐Whysall, K. , A. Soriano‐Arroquia , R. McCormick , C. Chinda , and B. McDonagh . 2020. “miR‐181a Regulates p62/SQSTM1, Parkin, and Protein DJ‐1 Promoting Mitochondrial Dynamics in Skeletal Muscle Aging.” Aging Cell 19, no. 4: e13140. 32291905 10.1111/acel.13140 PMC7189996

[cph470091-bib-0026] Groeneveld, K. 2024. “Muscle Physiology and Its Relations to the Whole Body in Health and Disease.” Acta Physiologica (Oxford, England) 240, no. 4: e14131. 38459776 10.1111/apha.14131

[cph470091-bib-0027] Guillot, S. J. , M. Bolborea , and L. Dupuis . 2021. “Dysregulation of Energy Homeostasis in Amyotrophic Lateral Sclerosis.” Current Opinion in Neurology 34, no. 5: 773–780. 34343139 10.1097/WCO.0000000000000982

[cph470091-bib-0028] Hao, L. Y. , B. I. Giasson , and N. M. Bonini . 2010. “DJ‐1 Is Critical for Mitochondrial Function and Rescues PINK1 Loss of Function.” Proceedings of the National Academy of Sciences of the United States of America 107, no. 21: 9747–9752. 20457924 10.1073/pnas.0911175107 PMC2906840

[cph470091-bib-0029] Hauser, D. N. , A. Mamais , M. M. Conti , et al. 2017. “Hexokinases Link DJ‐1 to the PINK1/Parkin Pathway.” Molecular Neurodegeneration 12, no. 1: 70. 28962651 10.1186/s13024-017-0212-x PMC5622528

[cph470091-bib-0030] Hayashi, T. , C. Ishimori , K. Takahashi‐Niki , et al. 2009. “DJ‐1 Binds to Mitochondrial Complex I and Maintains Its Activity.” Biochemical and Biophysical Research Communications 390, no. 3: 667–672. 19822128 10.1016/j.bbrc.2009.10.025

[cph470091-bib-0031] Hod, Y. , S. N. Pentyala , T. C. Whyard , and M. R. el‐Maghrabi . 1999. “Identification and Characterization of a Novel Protein That Regulates RNA‐Protein Interaction.” Journal of Cellular Biochemistry 72, no. 3: 435–444. 10022524

[cph470091-bib-0032] Howitz, K. T. , K. J. Bitterman , H. Y. Cohen , et al. 2003. “Small Molecule Activators of Sirtuins Extend *Saccharomyces cerevisiae* Lifespan.” Nature 425, no. 6954: 191–196. 12939617 10.1038/nature01960

[cph470091-bib-0033] Imberechts, D. , I. Kinnart , F. Wauters , et al. 2022. “DJ‐1 Is an Essential Downstream Mediator in PINK1/Parkin‐Dependent Mitophagy.” Brain 145, no. 12: 4368–4384. 36039535 10.1093/brain/awac313 PMC9762950

[cph470091-bib-0034] Inden, M. , Y. Kitamura , K. Takahashi , et al. 2011. “Protection Against Dopaminergic Neurodegeneration in Parkinson's Disease‐Model Animals by a Modulator of the Oxidized Form of DJ‐1, a Wild‐Type of Familial Parkinson's Disease‐Linked PARK7.” Journal of Pharmacological Sciences 117, no. 3: 189–203. 22041943 10.1254/jphs.11151fp

[cph470091-bib-0035] Irrcher, I. , H. Aleyasin , E. L. Seifert , et al. 2010. “Loss of the Parkinson's Disease‐Linked Gene DJ‐1 Perturbs Mitochondrial Dynamics.” Human Molecular Genetics 19, no. 19: 3734–3746. 20639397 10.1093/hmg/ddq288

[cph470091-bib-0036] Ito, G. , H. Ariga , Y. Nakagawa , and T. Iwatsubo . 2006. “Roles of Distinct Cysteine Residues in S‐Nitrosylation and Dimerization of DJ‐1.” Biochemical and Biophysical Research Communications 339, no. 2: 667–672. 16316629 10.1016/j.bbrc.2005.11.058

[cph470091-bib-0037] Janssen, I. , D. S. Shepard , P. T. Katzmarzyk , and R. Roubenoff . 2004. “The Healthcare Costs of Sarcopenia in the United States.” Journal of the American Geriatrics Society 52, no. 1: 80–85. 14687319 10.1111/j.1532-5415.2004.52014.x

[cph470091-bib-0038] Just, N. , C. Moreau , P. Lassalle , et al. 2007. “High Erythropoietin and Low Vascular Endothelial Growth Factor Levels in Cerebrospinal Fluid From Hypoxemic ALS Patients Suggest an Abnormal Response to Hypoxia.” Neuromuscular Disorders 17, no. 2: 169–173. 17142042 10.1016/j.nmd.2006.10.004

[cph470091-bib-0039] Kahle, P. J. , J. Waak , and T. Gasser . 2009. “DJ‐1 and Prevention of Oxidative Stress in Parkinson's Disease and Other Age‐Related Disorders.” Free Radical Biology & Medicine 47, no. 10: 1354–1361. 19686841 10.1016/j.freeradbiomed.2009.08.003

[cph470091-bib-0040] Kim, J. , K. J. Won , S. H. Jung , et al. 2015. “DJ‐1 Protects Against Undernutrition‐Induced Atrophy Through Inhibition of the MAPK‐Ubiquitin Ligase Pathway in Myoblasts.” Life Sciences 143: 50–57. 26408915 10.1016/j.lfs.2015.09.016

[cph470091-bib-0041] Kinumi, T. , J. Kimata , T. Taira , H. Ariga , and E. Niki . 2004. “Cysteine‐106 of DJ‐1 Is the Most Sensitive Cysteine Residue to Hydrogen Peroxide‐Mediated Oxidation In Vivo in Human Umbilical Vein Endothelial Cells.” Biochemical and Biophysical Research Communications 317, no. 3: 722–728. 15081400 10.1016/j.bbrc.2004.03.110

[cph470091-bib-0042] Kitamura, Y. , S. Watanabe , M. Taguchi , et al. 2011. “Neuroprotective Effect of a New DJ‐1‐Binding Compound Against Neurodegeneration in Parkinson's Disease and Stroke Model Rats.” Molecular Neurodegeneration 6, no. 1: 48. 21740546 10.1186/1750-1326-6-48 PMC3141555

[cph470091-bib-0043] Lev, N. , Y. Barhum , I. Lotan , I. Steiner , and D. Offen . 2015. “DJ‐1 Knockout Augments Disease Severity and Shortens Survival in a Mouse Model of ALS.” PLoS One 10, no. 3: e0117190. 25822630 10.1371/journal.pone.0117190 PMC4379040

[cph470091-bib-0044] Lev, N. , D. Ickowicz , Y. Barhum , E. Melamed , and D. Offen . 2009. “DJ‐1 Changes in G93A‐SOD1 Transgenic Mice: Implications for Oxidative Stress in ALS.” Journal of Molecular Neuroscience 38, no. 2: 94–102. 18712292 10.1007/s12031-008-9138-7

[cph470091-bib-0045] McCoy, M. K. , and M. R. Cookson . 2011. “DJ‐1 Regulation of Mitochondrial Function and Autophagy Through Oxidative Stress.” Autophagy 7, no. 5: 531–532. 21317550 10.4161/auto.7.5.14684 PMC3127213

[cph470091-bib-0046] Miller, D. W. , R. Ahmad , S. Hague , et al. 2003. “L166P Mutant DJ‐1, Causative for Recessive Parkinson's Disease, Is Degraded Through the Ubiquitin‐Proteasome System.” Journal of Biological Chemistry 278, no. 38: 36588–36595. 12851414 10.1074/jbc.M304272200

[cph470091-bib-0047] Mita, Y. , Y. Kataoka , Y. Saito , et al. 2018. “Distribution of Oxidized DJ‐1 in Parkinson's Disease‐Related Sites in the Brain and in the Peripheral Tissues: Effects of Aging and a Neurotoxin.” Scientific Reports 8, no. 1: 12056. 30104666 10.1038/s41598-018-30561-z PMC6089991

[cph470091-bib-0048] Moreau, C. , P. Gosset , J. Kluza , et al. 2011. “Deregulation of the Hypoxia Inducible Factor‐1α Pathway in Monocytes From Sporadic Amyotrophic Lateral Sclerosis Patients.” Neuroscience 172: 110–117. 20977930 10.1016/j.neuroscience.2010.10.040

[cph470091-bib-0049] Moscovitz, O. , G. Ben‐Nissan , I. Fainer , D. Pollack , L. Mizrachi , and M. Sharon . 2015. “The Parkinson's‐Associated Protein DJ‐1 Regulates the 20S Proteasome.” Nature Communications 6: 6609. 10.1038/ncomms7609 25833141

[cph470091-bib-0050] Moussa, C. E. , Q. Fu , P. Kumar , et al. 2006. “Transgenic Expression of Beta‐APP in Fast‐Twitch Skeletal Muscle Leads to Calcium Dyshomeostasis and IBM‐Like Pathology.” FASEB Journal 20, no. 12: 2165–2167. 16940437 10.1096/fj.06-5763fje

[cph470091-bib-0051] Nagakubo, D. , T. Taira , H. Kitaura , et al. 1997. “DJ‐1, a Novel Oncogene Which Transforms Mouse NIH3T3 Cells in Cooperation With Ras.” Biochemical and Biophysical Research Communications 231, no. 2: 509–513. 9070310 10.1006/bbrc.1997.6132

[cph470091-bib-0052] Neves, M. , M. Grãos , S. I. Anjo , and B. Manadas . 2022. “Modulation of Signaling Pathways by DJ‐1: An Updated Overview.” Redox Biology 51: 102283. 35303520 10.1016/j.redox.2022.102283 PMC8928136

[cph470091-bib-0053] Park, H. R. , and E. J. Yang . 2021. “Oxidative Stress as a Therapeutic Target in Amyotrophic Lateral Sclerosis: Opportunities and Limitations.” Diagnostics (Basel) 11, no. 9: 1546. 34573888 10.3390/diagnostics11091546 PMC8465946

[cph470091-bib-0054] Parsanejad, M. , Y. Zhang , D. Qu , et al. 2014. “Regulation of the VHL/HIF‐1 Pathway by DJ‐1.” Journal of Neuroscience 34, no. 23: 8043–8050. 24899725 10.1523/JNEUROSCI.1244-13.2014 PMC6608259

[cph470091-bib-0055] Powers, S. K. , and M. J. Jackson . 2008. “Exercise‐Induced Oxidative Stress: Cellular Mechanisms and Impact on Muscle Force Production.” Physiological Reviews 88, no. 4: 1243–1276. 18923182 10.1152/physrev.00031.2007 PMC2909187

[cph470091-bib-0056] Raninga, P. V. , G. Di Trapani , and K. F. Tonissen . 2017. “The Multifaceted Roles of DJ‐1 as an Antioxidant.” Advances in Experimental Medicine and Biology 1037: 67–87. 29147904 10.1007/978-981-10-6583-5_6

[cph470091-bib-0057] Roca‐Rivada, A. , O. al‐Massadi , C. Castelao , et al. 2012. “Muscle Tissue as an Endocrine Organ: Comparative Secretome Profiling of Slow‐Oxidative and Fast‐Glycolytic Rat Muscle Explants and Its Variation With Exercise.” Journal of Proteomics 75, no. 17: 5414–5425. 22800642 10.1016/j.jprot.2012.06.037

[cph470091-bib-0058] Rostad, K. O. , T. Trognitz , A. K. Frøyset , E. Bifulco , and K. E. Fladmark . 2024. “Accelerated Sarcopenia Phenotype in the DJ‐1/Park7‐Knockout Zebrafish.” Antioxidants (Basel) 13, no. 12: 1509. 39765837 10.3390/antiox13121509 PMC11673048

[cph470091-bib-0059] Sachdev, S. , and K. J. Davies . 2008. “Production, Detection, and Adaptive Responses to Free Radicals in Exercise.” Free Radical Biology & Medicine 44, no. 2: 215–223. 18191757 10.1016/j.freeradbiomed.2007.07.019

[cph470091-bib-0060] Sarlette, A. , K. Krampfl , C. Grothe , et al. 2008. “Nuclear Erythroid 2‐Related Factor 2‐Antioxidative Response Element Signaling Pathway in Motor Cortex and Spinal Cord in Amyotrophic Lateral Sclerosis.” Journal of Neuropathology and Experimental Neurology 67, no. 11: 1055–1062. 18957896 10.1097/NEN.0b013e31818b4906

[cph470091-bib-0061] Seyfarth, K. , G. Poschmann , J. Rozman , et al. 2015. “The Development of Diet‐Induced Obesity and Associated Metabolic Impairments in Dj‐1 Deficient Mice.” Journal of Nutritional Biochemistry 26, no. 1: 75–81. 25448609 10.1016/j.jnutbio.2014.09.002

[cph470091-bib-0062] Shi, S. Y. , S.‐Y. Lu , T. Sivasubramaniyam , et al. 2015. “DJ‐1 Links Muscle ROS Production With Metabolic Reprogramming and Systemic Energy Homeostasis in Mice.” Nature Communications 6: 7415. 10.1038/ncomms8415 PMC4490365 26077864

[cph470091-bib-0063] Shtifman, A. , C. W. Ward , D. R. Laver , et al. 2010. “Amyloid‐β Protein Impairs Ca2+ Release and Contractility in Skeletal Muscle.” Neurobiology of Aging 31, no. 12: 2080–2090. 19108934 10.1016/j.neurobiolaging.2008.11.003 PMC2901770

[cph470091-bib-0064] Shtifman, A. , N. Zhong , J. R. Lopez , J. Shen , and J. Xu . 2011. “Altered Ca^2+^ Homeostasis in the Skeletal Muscle of DJ‐1 Null Mice.” Neurobiology of Aging 32, no. 1: 125–132. 19683835 10.1016/j.neurobiolaging.2009.07.010 PMC2888942

[cph470091-bib-0065] Soares, P. , C. Silva , D. Chavarria , F. S. G. Silva , P. J. Oliveira , and F. Borges . 2023. “Drug Discovery and Amyotrophic Lateral Sclerosis: Emerging Challenges and Therapeutic Opportunities.” Ageing Research Reviews 83: 101790. 36402404 10.1016/j.arr.2022.101790

[cph470091-bib-0066] Solana‐Manrique, C. , F. J. Sanz , E. Ripollés , et al. 2020. “Enhanced Activity of Glycolytic Enzymes in Drosophila and Human Cell Models of Parkinson's Disease Based on DJ‐1 Deficiency.” Free Radical Biology & Medicine 158: 137–148. 32726690 10.1016/j.freeradbiomed.2020.06.036

[cph470091-bib-0067] Solana‐Manrique, C. , F. J. Sanz , I. Torregrosa , et al. 2022. “Metabolic Alterations in a Drosophila Model of Parkinson's Disease Based on DJ‐1 Deficiency.” Cells 11, no. 3: 331. 35159141 10.3390/cells11030331 PMC8834223

[cph470091-bib-0068] Song, I. K. , M. S. Kim , D. H. Shin , et al. 2021. “Stepwise Oxidations Play Key Roles in the Structural and Functional Regulations of DJ‐1.” Biochemical Journal 478, no. 19: 3505–3525. 34515295 10.1042/BCJ20210245 PMC8753860

[cph470091-bib-0069] Szelechowski, M. , N. Amoedo , E. Obre , et al. 2018. “Metabolic Reprogramming in Amyotrophic Lateral Sclerosis.” Scientific Reports 8, no. 1: 3953. 29500423 10.1038/s41598-018-22318-5 PMC5834494

[cph470091-bib-0070] Taira, T. , Y. Saito , T. Niki , S. M. Iguchi‐Ariga , K. Takahashi , and H. Ariga . 2004. “DJ‐1 Has a Role in Antioxidative Stress to Prevent Cell Death.” EMBO Reports 5, no. 2: 213–218. 14749723 10.1038/sj.embor.7400074 PMC1298985

[cph470091-bib-0071] Taira, T. , K. Takahashi , R. Kitagawa , S. M. M. Iguchi‐Ariga , and H. Ariga . 2001. “Molecular Cloning of Human and Mouse DJ‐1 Genes and Identification of Sp1‐Dependent Activation of the Human DJ‐1 Promoter.” Gene 263, no. 1–2: 285–292. 11223268 10.1016/s0378-1119(00)00590-4

[cph470091-bib-0072] Takahashi‐Niki, K. , Y. Ganaha , T. Niki , et al. 2016. “DJ‐1 Activates SIRT1 Through Its Direct Binding to SIRT1.” Biochemical and Biophysical Research Communications 474, no. 1: 131–136. 27105916 10.1016/j.bbrc.2016.04.084

[cph470091-bib-0073] Tanti, G. K. , and S. K. Goswami . 2014. “SG2NA Recruits DJ‐1 and Akt Into the Mitochondria and Membrane to Protect Cells From Oxidative Damage.” Free Radical Biology & Medicine 75: 1–13. 25035075 10.1016/j.freeradbiomed.2014.07.009

[cph470091-bib-0074] Tao, X. , and L. Tong . 2003. “Crystal Structure of Human DJ‐1, a Protein Associated With Early Onset Parkinson's Disease.” Journal of Biological Chemistry 278, no. 33: 31372–31379. 12761214 10.1074/jbc.M304221200

[cph470091-bib-0075] Terracciano, C. , A. Nogalska , W. K. Engel , S. Wojcik , and V. Askanas . 2008. “In Inclusion‐Body Myositis Muscle Fibers Parkinson‐Associated DJ‐1 Is Increased and Oxidized.” Free Radical Biology & Medicine 45, no. 6: 773–779. 18601999 10.1016/j.freeradbiomed.2008.05.030 PMC2579266

[cph470091-bib-0076] Trist, B. G. , S. Genoud , S. Roudeau , et al. 2022. “Altered SOD1 Maturation and Post‐Translational Modification in Amyotrophic Lateral Sclerosis Spinal Cord.” Brain 145, no. 9: 3108–3130. 35512359 10.1093/brain/awac165 PMC9473357

[cph470091-bib-0077] Wang, F. , Y. Lu , F. Qi , et al. 2014. “Effect of the Human SOD1‐G93A Gene on the Nrf2/ARE Signaling Pathway in NSC‐34 Cells.” Molecular Medicine Reports 9, no. 6: 2453–2458. 24682253 10.3892/mmr.2014.2087

[cph470091-bib-0078] Wang, T. , Y. Xue , Y. Li , et al. 2022. “DJ‐1 Protein Inhibits Apoptosis in Cerebral Ischemia by Regulating the Notch1 and Nuclear Factor Erythroid2‐Related Factor 2 Signaling Pathways.” Neuroscience 504: 33–46. 36167256 10.1016/j.neuroscience.2022.09.016

[cph470091-bib-0079] Wang, X. , T. G. Petrie , Y. Liu , J. Liu , H. Fujioka , and X. Zhu . 2012. “Parkinson's Disease‐Associated DJ‐1 Mutations Impair Mitochondrial Dynamics and Cause Mitochondrial Dysfunction.” Journal of Neurochemistry 121, no. 5: 830–839. 22428580 10.1111/j.1471-4159.2012.07734.x PMC3740560

[cph470091-bib-0080] Wang, Z. , J. Liu , S. Chen , et al. 2011. “DJ‐1 Modulates the Expression of cu/Zn‐Superoxide Dismutase‐1 Through the Erk1/2‐Elk1 Pathway in Neuroprotection.” Annals of Neurology 70, no. 4: 591–599. 21796667 10.1002/ana.22514

[cph470091-bib-0081] Wang, Z. Y. , J. Cheng , B. Liu , et al. 2021. “Protein Deglycase DJ‐1 Deficiency Induces Phenotypic Switching in Vascular Smooth Muscle Cells and Exacerbates Atherosclerotic Plaque Instability.” Journal of Cellular and Molecular Medicine 25, no. 6: 2816–2827. 33501750 10.1111/jcmm.16311 PMC7957272

[cph470091-bib-0082] Wilson, M. A. 2011. “The Role of Cysteine Oxidation in DJ‐1 Function and Dysfunction.” Antioxidants & Redox Signaling 15, no. 1: 111–122. 20812780 10.1089/ars.2010.3481 PMC3110098

[cph470091-bib-0083] Wilson, M. A. , C. V. S. Amour , J. L. Collins , D. Ringe , and G. A. Petsko . 2004. “The 1.8‐A Resolution Crystal Structure of YDR533Cp From *Saccharomyces cerevisiae*: A Member of the DJ‐1/ThiJ/PfpI Superfamily.” Proceedings of the National Academy of Sciences of the United States of America 101, no. 6: 1531–1536. 14745011 10.1073/pnas.0308089100 PMC341769

[cph470091-bib-0084] Wilson, M. A. , J. L. Collins , Y. Hod , D. Ringe , and G. A. Petsko . 2003. “The 1.1‐A Resolution Crystal Structure of DJ‐1, the Protein Mutated in Autosomal Recessive Early Onset Parkinson's Disease.” Proceedings of the National Academy of Sciences of the United States of America 100, no. 16: 9256–9261. 12855764 10.1073/pnas.1133288100 PMC170905

[cph470091-bib-0085] Xu, S. , X. Yang , Y. Qian , and Q. Xiao . 2018. “Parkinson's Disease‐Related DJ‐1 Modulates the Expression of Uncoupling Protein 4 Against Oxidative Stress.” Journal of Neurochemistry 145, no. 4: 312–322. 29315581 10.1111/jnc.14297

[cph470091-bib-0086] Yamaguchi, H. , and J. Shen . 2007. “Absence of Dopaminergic Neuronal Degeneration and Oxidative Damage in Aged DJ‐1‐Deficient Mice.” Molecular Neurodegeneration 2: 10. 17535435 10.1186/1750-1326-2-10 PMC1891294

[cph470091-bib-0087] Yan, Y. F. , W. J. Yang , Q. Xu , et al. 2015. “DJ‐1 Upregulates Anti‐Oxidant Enzymes and Attenuates Hypoxia/Re‐Oxygenation‐Induced Oxidative Stress by Activation of the Nuclear Factor Erythroid 2‐Like 2 Signaling Pathway.” Molecular Medicine Reports 12, no. 3: 4734–4742. 26081287 10.3892/mmr.2015.3947

[cph470091-bib-0088] Yanagida, T. , Y. Kitamura , K. Yamane , et al. 2009. “Protection Against Oxidative Stress‐Induced Neurodegeneration by a Modulator for DJ‐1, the Wild‐Type of Familial Parkinson's Disease‐Linked PARK7.” Journal of Pharmacological Sciences 109, no. 3: 463–468. 19276614 10.1254/jphs.08323sc

[cph470091-bib-0089] Yang, C. C. , R. B. Alvarez , W. K. Engel , and V. Askanas . 1996. “Increase of Nitric Oxide Synthases and Nitrotyrosine in Inclusion‐Body Myositis.” Neuroreport 8, no. 1: 153–158. 9051771 10.1097/00001756-199612200-00031

[cph470091-bib-0090] Yang, J. , M. J. Kim , W. Yoon , et al. 2017. “Isocitrate Protects DJ‐1 Null Dopaminergic Cells From Oxidative Stress Through NADP+‐Dependent Isocitrate Dehydrogenase (IDH).” PLoS Genetics 13, no. 8: e1006975. 28827794 10.1371/journal.pgen.1006975 PMC5578699

[cph470091-bib-0091] Yu, H. , J. N. Waddell , S. Kuang , and C. A. Bidwell . 2014. “Park7 Expression Influences Myotube Size and Myosin Expression in Muscle.” PLoS One 9, no. 3: e92030. 24637782 10.1371/journal.pone.0092030 PMC3956870

[cph470091-bib-0092] Zhang, L. , M. Shimoji , B. Thomas , et al. 2005. “Mitochondrial Localization of the Parkinson's Disease Related Protein DJ‐1: Implications for Pathogenesis.” Human Molecular Genetics 14, no. 14: 2063–2073. 15944198 10.1093/hmg/ddi211

[cph470091-bib-0093] Zhang, S. , H. Yan , J. Ding , et al. 2023. “Skeletal Muscle‐Specific DJ‐1 Ablation‐Induced Atrogenes Expression and Mitochondrial Dysfunction Contributing to Muscular Atrophy.” Journal of Cachexia, Sarcopenia and Muscle 14, no. 5: 2126–2142. 37469245 10.1002/jcsm.13290 PMC10570112

[cph470091-bib-0094] Zhang, Y. , X. G. Gong , Z. Z. Wang , et al. 2016. “Overexpression of DJ‐1/PARK7, the Parkinson's Disease‐Related Protein, Improves Mitochondrial Function via Akt Phosphorylation on Threonine 308 in Dopaminergic Neuron‐Like Cells.” European Journal of Neuroscience 43, no. 10: 1379–1388. 26913805 10.1111/ejn.13216

[cph470091-bib-0095] Zhang, Y. , X. R. Li , L. Zhao , G. L. Duan , L. Xiao , and H. P. Chen . 2018. “DJ‐1 Preserving Mitochondrial Complex I Activity Plays a Critical Role in Resveratrol‐Mediated Cardioprotection Against Hypoxia/Reoxygenation‐Induced Oxidative Stress.” Biomedicine & Pharmacotherapy 98: 545–552. 29287203 10.1016/j.biopha.2017.12.094

[cph470091-bib-0096] Zhao, J. , X. Wang , Z. Huo , et al. 2022. “The Impact of Mitochondrial Dysfunction in Amyotrophic Lateral Sclerosis.” Cells 11, no. 13: 2049. 35805131 10.3390/cells11132049 PMC9265651

[cph470091-bib-0097] Zhao, N. , T. Wang , L. Peng , Y. Li , Y. Zhao , and S. Yu . 2021. “Attenuation of Inflammation by DJ‐1 May be a Drug Target for Cerebral Ischemia‐Reperfusion Injury.” Neurochemical Research 46, no. 6: 1470–1479. 33683631 10.1007/s11064-021-03288-z

[cph470091-bib-0098] Zheng, H. , C. Zhou , X. Lu , et al. 2018. “DJ‐1 Promotes Survival of Human Colon Cancer Cells Under Hypoxia by Modulating HIF‐1α Expression Through the PI3K‐AKT Pathway.” Cancer Management and Research 10: 4615–4629. 30410397 10.2147/CMAR.S172008 PMC6199970

[cph470091-bib-0099] Zhou, W. , J. C. Barkow , and C. R. Freed . 2017. “Running Wheel Exercise Reduces α‐Synuclein Aggregation and Improves Motor and Cognitive Function in a Transgenic Mouse Model of Parkinson's Disease.” PLoS One 12, no. 12: e0190160. 29272304 10.1371/journal.pone.0190160 PMC5741244

[cph470091-bib-0100] Zhou, Y. , X. Zhang , J. S. Baker , G. W. Davison , and X. Yan . 2024. “Redox Signaling and Skeletal Muscle Adaptation During Aerobic Exercise.” iScience 27, no. 5: 109643. 38650987 10.1016/j.isci.2024.109643 PMC11033207

